# Parenting strategies for reducing adolescent alcohol use: a Delphi consensus study

**DOI:** 10.1186/1471-2458-11-13

**Published:** 2011-01-06

**Authors:** Siobhan M Ryan, Anthony F Jorm, Claire M Kelly, Laura M Hart, Amy J Morgan, Dan I Lubman

**Affiliations:** 1 Orygen Youth Health Research Centre, Centre for Youth Mental Health, University of Melbourne, Victoria, Australia; 2 Turning Point Alcohol and Drug Centre, Eastern Health and Monash University, Victoria, Australia

## Abstract

**Background:**

International concern regarding the increase in preventable harms attributed to adolescent alcohol consumption has led to growing political and medical consensus that adolescents should avoid drinking for as long as possible. For this recommendation to be adopted, parents and guardians of adolescents require information about strategies that they can employ to prevent or reduce their adolescent's alcohol use that are supported by evidence.

**Methods:**

The Delphi method was used to obtain expert consensus on parenting strategies effective in preventing and reducing adolescent alcohol consumption. A literature search identified 457 recommendations for parents to reduce their adolescent child's alcohol use. These recommendations were presented to a panel of 38 Australian experts who were asked to rate their importance over three survey rounds.

**Results:**

There were 289 parenting strategies that were endorsed as important or essential in reducing adolescent alcohol use by ≥90% of the panel. These strategies were categorised into 11 sub-headings: things parents should know about adolescent alcohol use, delaying adolescent's introduction to alcohol, modelling responsible drinking and attitudes towards alcohol, talking to adolescents about alcohol, establishing family rules, monitoring adolescents when unsupervised, preparing adolescents for peer pressure, unsupervised adolescent drinking, what to do when an adolescent has been drinking without parental permission, hosting adolescent parties, and establishing and maintaining a good parent-child relationship. The endorsed strategies were written into a document suitable for parents.

**Conclusions:**

A comprehensive set of parenting strategies for preventing or reducing adolescent alcohol consumption were identified. These strategies can be promoted to parents to help them implement national recommendations for use of alcohol by young people.

## Background

There has been growing international concern regarding the increase in preventable harms attributed to adolescent alcohol consumption [1]. Such concern has prompted medical and political debate regarding the delaying of adolescent alcohol consumption for as long as possible [2,3]. Young drinkers are more likely to drink at risky levels [4], placing themselves at an increased risk of serious physical injury and unsafe or unwanted sex [5-7]. Furthermore, alcohol consumption is associated with increased adolescent mortality [6,8], and alcohol consumption at a young age (i.e. before the age of 15) increases the risk of developing alcohol-related problems later in life [9-12]. Encouraging adolescents to avoid alcohol is now embedded within the new national guidelines for alcohol consumption in both Australia and the United Kingdom (UK). Both guidelines recommend that people under the age of 18 delay initiating alcohol consumption for as long as possible, and that people under the age of 15 not drink any alcohol at all [2,3]. For these recommendations to be widely adopted, a cultural shift is required within both nations, particularly as experimentation with alcohol has been typically viewed as a normal and relatively benign stage of adolescence [13,14].

Both the *Australian Guidelines to Reduce Health Risks from Drinking Alcohol *published by the National Health and Medical Research Council and the *Guidance on the Alcohol Consumption by Children and Young People *from the Chief Medical Officer of the United Kingdom highlight the role of parents and carers in implementing recommendations for young people regarding alcohol consumption [2,3]. However, neither guideline provides parents and carers with advice on how they are to do this. Parents and guardians attempting to implement these recommendations may encounter resistance, as most young people view alcohol consumption as a positive experience, with few negative consequences [7,14]. If these new guidelines are to be put into practice, parents need access to information about what parenting strategies are likely to be effective in preventing or reducing their adolescent's alcohol consumption.

Longitudinal studies investigating factors associated with adolescent alcohol use have identified a number of parenting variables as influential in delaying adolescent alcohol initiation and reducing consequent alcohol use. These include: parental modelling, provision of alcohol, alcohol-specific communication, parental disapproval of drinking, parental discipline, rules about alcohol, parental monitoring and the quality of the parent-child relationship (including the level of conflict between the parent and the child, parental support, parental involvement, and the level and quality of communication between the parent and the child) [15]. Although these variables provide general guidance on parenting styles that are influential in reducing adolescent alcohol use, they do not clearly describe specific parenting strategies that can be readily put into practice. For this literature to be informative for parents, the parenting styles identified need to be made more explicit as individual, actionable parenting strategies.

The present study aimed to produce specific guidance for parents on actions they can take to reduce alcohol use in their adolescent children. The Delphi methodology was used to establish expert consensus on strategies for parents that are effective in preventing or reducing their adolescent child's alcohol consumption, in line with recommendations outlined by the new Australian and UK alcohol guidelines for young people. The developed strategies could be promoted to the general public with the ultimate goal of reducing levels of preventable harms associated with adolescent alcohol use. The strategies could also provide the foundation for interventions (e.g. websites, public information campaigns, parent training programs) to help parents prevent their children from consuming alcohol in excess of national guideline recommendations.

## Methods

The Delphi method involves a panel of experts independently and privately providing their level of agreement with a series of statements [16]. There are many variations on this method. The approach used here involved reviewing sources of advice for parents and constructing a set of statements from this literature. These statements were presented to the panel of experts in three sequential rounds. Members of the expert panel were also given the opportunity to provide feedback and make suggestions for additional statements in the first round. New ideas identified by panel member comments were included as statements in the second round for the whole panel to evaluate. To help achieve consensus, a summary of the whole panel's responses was provided to each panel member after each round. Panel members then had the option to change or maintain their original rating of statements in subsequent rounds. The outcome of this process was a list of statements for which there was substantial consensus in ratings (>90%).

### Panel formation

The panel was made up of experts with a minimum of five years experience in one or more of the following areas: the development and delivery of alcohol and drug education to adolescents and/or parents (education experts), research investigating adolescent alcohol use and parenting practices (research experts), or the clinical treatment of adolescents who have experienced alcohol use disorders (clinical experts). Education experts were identified via a contact list of drug education officers obtained from the Australian Department of Education, Employment and Workplace Relations. Research experts were recruited by contacting the major national drug and alcohol research organisations in Australia. Clinical experts were recruited by contacting professional groups and organisations associated with the treatment of alcohol and other drugs in young people. People known to the authors as having relevant clinical or research experience were also invited to participate.

Experts were recruited throughout Australia via an email invitation designed to solicit interest from potential panel members. This invitation included eligibility criteria for expert panel participation and a hyperlink to an online description of the study which included an outline of the contribution required by panel members and the voluntary nature of participation. Everyone who received the invitation was encouraged to discuss it with anyone else who might be interested in participating on the expert panel and met eligibility criteria. Informed consent was implied by responding to the online questionnaire. This project was granted human research ethics committee approval by the University of Melbourne.

### Questionnaire development and administration

A systematic search of websites and electronic information brochures, leaflets and hand-outs from service providers or information centres was carried out to identify the full range of strategies recommended to parents to prevent or reduce their adolescent's alcohol consumption. These sources were identified via a systematic internet search using five search engines (http://google.com, http://google.ca, http://google.com.au, http://google.co.nz, http://google.co.uk) with the search term "Parenting to reduce adolescent alcohol use". The top 50 websites produced by these searches were examined for all recommendations for parents to prevent their adolescent child from misusing alcohol. Relevant links appearing on these top 50 websites were also examined for applicable parenting strategies. Books and hardcopies of leaflets and pamphlets focussed on parenting to prevent adolescent alcohol use were also examined for parenting recommendations. Academic literature was also searched for relevant parenting strategies via seven electronic databases (Academic Search Complete, Family Studies Abstracts, Medline, PsycARTICLES, Psychology and Behavioral Sciences Collection, PsycINFO, and Social Work Abstracts) using the search terms parenting AND adolescent OR youth AND alcohol. The academic literature did not contribute any strategies included in the surveys, however it did produce the evidence for the reviews provided to panel members described later.

The literature search found 1864 recommendations for parents to prevent their child from misusing alcohol from 80 sources. A large number of these items were repetitive in content (though worded differently), and many items combined multiple recommendations in a single statement. In order to simplify the set of items, one author (SMR) grouped similar items together and identified the item that most succinctly expressed the idea contained within these grouped items. When no single item was clear and concise, this author wrote new items to best represent the idea discussed within the grouped items. This author also broke down statements discussing more than one idea into multiple items. Grouped items and new items were presented to a working group of five researchers who have extensive experience in Delphi research [17-22]. This working group was responsible for ensuring that all ideas within the statements identified in the literature search were represented, that items were not repetitive, that items were clear and that they were actionable by parents. Some items were edited by the working group to achieve these aims and to create consistency of wording across items. This process resulted in 457 items describing specific parenting strategies for inclusion in the first round questionnaire.

Items were organised into 22 sub-headings: things parents should know, parental modelling, delaying initiation, providing alcohol, talking to adolescents about alcohol, expressing disapproval, school and community resources, general parental discipline, alcohol-specific rules, consequences for when rules are broken, supervision and monitoring, parent-child relationship quality, family conflict, parental support, parental involvement, general communication, dealing with peer influence, preparation for situations involving alcohol, activities, community action, parties, and when an adolescent has been drinking without permission. Panel members were provided with a systematic review of relevant longitudinal research to consider in their ratings, for sections in the questionnaire where this was available (see [15] for an abridged version of this review). Table 1 shows example items from each sub-heading, as well as the sub-headings accompanied by an evidence review.

**Table 1:** Examples of parenting strategies under the sub-headings used in the Delphi surveys and the final document

Survey sub-headings	Final document sub-headings	Example parenting strategies
Things parents should know	Some things you should know about adolescent drinking	Parents should be aware that adolescents have less physical tolerance to the effects of alcohol
Delaying initiation*	Delay your adolescent's introduction to drinking *(including items from 'Provision of alcohol')*	Parents should be aware that the longer their adolescent delays alcohol use, the less likely they are to develop problems associated with alcohol.
Providing alcohol*		Parents should be aware that they can teach responsible drinking to their adolescent without allowing the adolescent to drink.
Parental modelling*	Model responsible drinking and attitudes towards alcohol	Parents who drink should model responsible drinking by never drinking and driving.
Talking to adolescents about alcohol*	Talk to your adolescent about alcohol *(including some items from 'Expressing disapproval' and 'School and community resources')*	When talking to their adolescent child about alcohol, parents should teach them that the effects of alcohol vary between individuals, depending upon the amount of alcohol, the person and the context.
Expressing disapproval*		Parents should not present a permissive approach to alcohol, as this can increase the likelihood of alcohol misuse by their adolescent child.
School and community resources		Parents should be aware of how alcohol is addressed in their adolescent's school curriculum.
General parental discipline*	Establish family rules *(including items from 'Alcohol-specific rules', and 'Consequences for when rules are broken')*	When establishing family rules parents should involve the adolescent in their development.
Alcohol-specific rules		In establishing family rules regarding alcohol, parents should ensure the adolescent knows that these rules are a protective measure, and not just a restriction on their freedom.
Consequences for when rules are broken		When establishing consequences for when family rules are broken, parents should make them very clear to their adolescent child.
Supervision and monitoring*	Monitor your adolescent when you are not around	Parents should be aware that parental monitoring reduces the likelihood of their adolescent misusing alcohol. Parents should monitor their adolescent by asking them where they will be when they are unsupervised.
Activities		Parents should be aware that adolescents who participate in activities that complement their interests and abilities are less likely to misuse alcohol.
Community Action		Parents should become involved in community activities aimed at the prevention of adolescent alcohol misuse.
Dealing with peer influence	Prepare your adolescent for peer pressure	Parents should be aware that if their adolescent's friends use alcohol, their adolescent is more likely to use alcohol.
Preparation for situations involving alcohol	Unsupervised adolescent drinking	Parents should discuss with their adolescent situations they may be faced with where they are pressured to drink to ensure they are sufficiently prepared for handling these situations.
When an adolescent has been drinking without permission	When your adolescent has been drinking without permission	If their adolescent comes home drunk, parents should wait until the adolescent is sober before talking to them about their behaviour.
Parties	Adolescent parties at your house	When hosting an adolescent party, parents should work with their adolescent to plan age appropriate activities to take the focus off drinking at the party.
Parent-child relationship quality*	Establish and maintain a good relationship with your adolescent child (*including items from 'Family conflict', 'Parental support', 'Parental involvement', and 'General Communication')*	Parents should praise their adolescent for their efforts as well as their achievements.
Family conflict*		Parents should not tease their adolescent in a way that could be perceived as hurtful.
Parental support*		Parents should ensure that their positive comments outweigh their negative comments in their interactions with their adolescent.
Parental involvement*		Parents can be involved with their adolescent by establishing a regular weekly routine for doing something special with the adolescent.
General communication*		Parents should encourage communication with their adolescent by asking the adolescent about topics that interest them, and listening to them when they talk.

* Denotes accompanied by evidence review

Panel members were asked to rate the importance of items for inclusion in recommended strategies for parents to prevent or reduce adolescent alcohol consumption. The rating scale was 1 = Essential, 2 = Important, 3 = Don't know/Depends, 4 = Unimportant, 5 = Should not be included. Panel members were asked to base ratings on whatever source of knowledge they had available to them, including research evidence, expertise in the clinical treatment of adolescents with alcohol problems, and/or expertise in teaching adolescents and/or parents how to avoid alcohol related problems during adolescence. In the questionnaire, *parenting strategies to prevent or reduce adolescent alcohol use *were defined as any parenting variable that a parent could actively modify with the potential to influence their adolescent's alcohol use.

Panel members were given the opportunity to provide feedback on items and suggest strategies not included in the questionnaire in Round 1. Suggestions judged by the working group to be a new idea were drafted into items and included in Round 2. Where is was apparent that items from Round 1 lacked clarity or confused panel members, new items were drafted for inclusion in Round 2. No new items were introduced in Round 3.

Three online questionnaires were sent to panel members using SurveyMonkey survey software (using SurveyMonkey, http://www.surveymonkey.com). They were given 6 weeks to complete Round 1, 3 weeks to complete Round 2, and 3 weeks to complete Round 3. Non-responders were sent up to two email reminders for each round.

### Statistical Analysis

The panel's responses to the questionnaire were analysed by calculating group percentages. Items from each round that were rated as either important or essential by 90% or more of the panel were endorsed as recommended strategies. Items rated as important or essential by less than 80% of the panel were not included as recommended strategies, and items rated as important or essential by between 80 and 89% of the panel were included in the subsequent round for re-rating. To analyse panel member's comments made in Round 1, one author (SMR) read through all the comments, grouped them together into common themes, identified new ideas presented within the comments, and drafted new items based on the grouped comments. Comments and newly drafted items were then presented to the working group to ensure all new ideas were represented. The working group edited these new items for Round 2 to ensure clarity and consistency of expression.

## Results

Thirty-eight panel members participated in Round 1 (13 male, 20 female). Of these participants, 18 identified their primary area of expertise as treatment, 13 as education and 7 as research. Participants were recruited from across Australia (14 from Victoria, 9 from New South Wales, 6 from Queensland, 4 from Western Australia, 3 from South Australia and 1 from Tasmania) plus 1 from New Zealand (who fulfilled the criterion of having more than 5 years of relevant experience in Australia). The Round 2 survey was completed by 34 of the Round 1 participants (89%), and Round 3 was completed by 31 of the Round 2 participants (82% of Round 1 participants).

### Round 1

**Figure 1:**
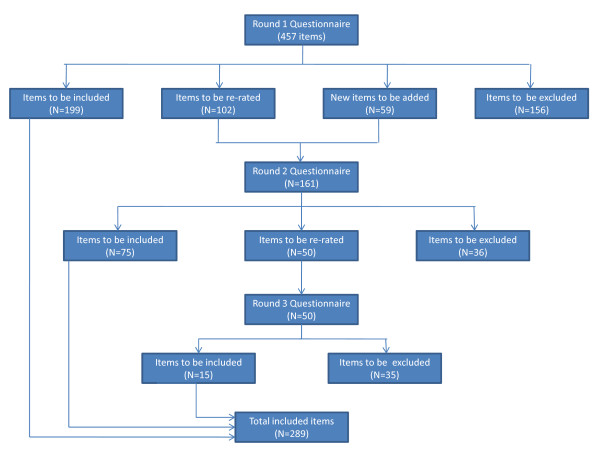
**The number of items that were included, re-rated and rejected in the 3 rounds of the study**.

Figure 1 illustrates the numbers of items that were included, excluded or re-rated during each round of questionnaires. In the Round 1 survey, 199 items were rated as essential or important by ≥90% of the expert panel, 188 items were excluded and 102 items met criteria to be re-rated in Round 2. Forty six new items were developed from panel members' suggestions in Round 1, and 13 new items were developed in order to clarify potential confusion regarding some Round 1 items, giving a total of 59 new items for inclusion in the Round 2 questionnaire.

### Round 2

Of the 161 items in Round 2, 75 achieved sufficient consensus to be included as a recommended strategy, and 36 were excluded. Fifty items reached the consensus level required to be re-rated in the third and final round.

### Final recommendations

In Round 3, 15 of the 50 items achieved sufficient consensus from panel members to be included as a recommended strategy. Items not endorsed in Round 3 were discarded. This made a total of 289 items to be included as recommended strategies for parents to prevent or reduce their adolescent child's alcohol consumption. See Additional File 1 for a full list of the items endorsed and rejected for each round.

A post-hoc analysis was performed to identify items judged by over 50% of the panel as 'unimportant' or 'should not be included'. Only two items were identified: "If their adolescent is over 15 and wants to drink at parties, parents should provide the adolescent with alcohol when they go to parties and gatherings, so that they can monitor how much the adolescent drinks", and "Parents should seek advice from other parents if unsure as to whether or not to supply alcohol to the adolescent for a party or gathering."

The 289 recommended strategies were synthesised into cohesive prose by one author (SMR) under sub-headings from the questionnaire (some sub-headings were discarded if there were too few items endorsed by the expert panel). This process required some editing to remove contextual strings from items and add conjunctions, however the content of items was not altered. The constructed sub-headings of text were then presented to the working group who organised them into a coherent order to produce a single document. The sub-headings included in the final document are shown in Table 1. This document was edited by the working group to ensure it was cohesive and comprehensible, and faithful to the items endorsed by the expert panel. The working group also confirmed that all recommendations endorsed by the expert panel were represented within the document. The draft document was then returned to members of the expert panel who were given the opportunity to provide feedback on its wording and layout. Panel member comments judged by the working group as improving the comprehensibility of the draft recommended strategies, without introducing a new idea, were integrated into the final document. See Additional File 2 to view this final document.

## Discussion

This study aimed to use expert consensus to identify parenting strategies effective in preventing or reducing adolescent alcohol consumption, in line with new Australian and UK guidelines. While there are many recommendations available to parents on how they can limit their adolescent's alcohol consumption, supporting references are rare and are likely to be based on personal opinion or interpretation of parenting variables identified in the academic literature.

This study identified 289 strategies that were categorised thematically into 11 sub-headings. Some of these sub-headings corresponded to parenting variables identified from longitudinal studies as influential in delaying and reducing adolescent alcohol consumption, while the remaining sub-headings concerned strategies that would be difficult to investigate in a longitudinal study. These newly identified sub-headings were 'Prepare your adolescent for peer pressure', 'Unsupervised adolescent drinking', 'When your adolescent has been drinking without permission', and 'Adolescent parties at your house'. Although the influence of peers has been researched (and even compared to the influence of parents in numerous studies), we were unable to find any studies investigating how parents should deal with the influence of peers on their adolescent's alcohol use [15].

It is noteworthy that when the reviews provided to panel members found no evidence that certain parenting variables were associated with adolescent alcohol use, corresponding parenting strategies were nevertheless often endorsed. This was particularly true of strategies under the sub-headings 'Talking to the adolescent about alcohol' and 'Alcohol-specific rules'. Such findings indicate that panel members used their own experience and expertise, and not only the evidence from longitudinal studies, which is a benefit provided by the Delphi consensus method.

The results of this study, as well as the findings of longitudinal research, identified many parenting variables that were unrelated to alcohol regulation as influential in delaying and reducing adolescent alcohol consumption. Examples include parenting strategies relating to the quality of the parent-adolescent relationship, such as parental support, involvement, and general communication. These findings suggest that while alcohol-specific strategies such as talking to an adolescent about the dangers of alcohol and establishing rules prohibiting alcohol consumption are important, it is also essential that parents invest in the quality of their relationship with their adolescent if they are to delay the adolescent's introduction to alcohol and influence their subsequent level of alcohol use.

There is debate in the lay literature about whether parents should introduce alcohol to their children in the family home in an attempt to teach the adolescent responsible attitudes and behaviours regarding alcohol consumption. No strategies involving parents providing adolescents with alcohol were endorsed by the expert panel, and suggestions regarding providing alcohol to adolescents when they go to parties were strongly rejected. However, items explicitly stating that parents should not provide their adolescent with alcohol were also not endorsed by the expert panel. Thus, while parents are strongly discouraged from providing their adolescent child with any alcohol, they are afforded some flexibility in choosing an approach that suits their individual situation.

A limitation of this study is that strategies identified may only be appropriate for Australian families, as the experts on the panel were solely from Australia. Further, the current study did not identify parenting strategies that might be relevant for families from culturally and linguistically diverse minority groups within the Australia. These limitations could be addressed by replication with panels from other relevant communities. The absence of parents of adolescents on the expert panel might also be considered a limitation. However, this was a deliberate decision on our part. In deciding what expertise would be required by panel members to best evaluate strategies for the majority of families, we believed that it would be necessary for panel members to have experience in dealing with a wide range of families, rather than experience that was limited to one individual family. We also considered it necessary that panel members have the expertise to understand the scientific research reviews that were provided to assist their judgements. As it was, comments made in Round 1 indicated that panel members did draw on their own personal experiences of parenting when identifying strategies to prevent or reduce adolescent alcohol.

In order to determine whether the parenting strategies identified in this study are practical and effective in preventing adolescent alcohol misuse, further research is required. In this regard, we have developed a web-based parenting intervention based on the strategies identified http://www.parentingstrategies.net, and will evaluate its efficacy via a randomised controlled trial.

Despite some limitations, the current study has produced recommendations for parents on specific strategies they can use to help prevent or reduce their adolescent's alcohol consumption. In doing so, we hope that these strategies provide practical support to parents in implementing the new Australian and UK recommendations for alcohol consumption by young people.

## Conclusions

A comprehensive set of parenting strategies for preventing or reducing adolescent alcohol consumption was identified via expert consensus. These strategies can be promoted to parents to help them implement national recommendations for use of alcohol by young people.

## Competing interests

The authors declare that they have no competing interests.

## Authors' contributions

AFJ and DIL designed the study and wrote the protocol. SMR completed the literature review, constructed the initial survey, recruited participants, collected and analysed the data, and prepared drafts of the guidelines. A working group, consisting of AFJ, DIL, CMK, LMH and AJM gathered regularly to give feedback and make improvements on each draft of the Rounds 1, 2 and 3 surveys and the final guidelines. SMR wrote the first draft of the manuscript with input from DIL and AFJ. All authors contributed to and have approved the final manuscript.

## Pre-publication history

The pre-publication history for this paper can be accessed here:

http://www.biomedcentral.com/1471-2458/11/13/prepub

## Supplementary Material

Additional file 1 **Table S1**. Items endorsed and rejected from the 3 rounds of the study. Click here for file

Additional file 2 **Parenting guideline for adolescent alcohol use**. Final document of parenting strategies for preventing or reducing adolescent alcohol use. Click here for file

## References

[B1] Livingston M Recent trends in risky alcohol consumption and related harm among young people in Victoria, Australia Aust N Z J Public Health 2008 32 266 271 10.1111/j.1753-6405.2008.00227.x 18578827

[B2] Donaldson L Guidance on the Consumption of Alcohol by Children and Young People. A report by the Chief Medical Officer 2009 Department of Health http://www.dh.gov.uk/en/Publicationsandstatistics/Publications/PublicationsPolicyAndGuidance/DH_110258

[B3] National Health and Medical Research Council Australian Guidelines to Reduce Health Risks from Drinking Alcohol 2009 Canberra

[B4] Fuller EE Drug use, smoking and drinking among young people in England in 2007 2007 Health and Social Care Information Centre. London

[B5] Bonomo Y Coffey C Wolfe R Lynskey M Bowes G Patton G Adverse outcomes of alcohol use in adolescents Addiction 2001 96 1485 1496 10.1046/j.1360-0443.2001.9610148512.x 11571067

[B6] Chikritzhs T Pascal R Jones P Under-Age Drinking Among 14-17 Year Olds and Related Harms in Australia 2004 Perth (AUST): National Drug Institute

[B7] Hibell B Guttormsson U Ahlstrom T Balakireva O Bjarnason T Kokkevi K Kraus L The 2007 ESPAD Report: Substance Use Amongst Students in 35 European Countries 2009 Stockholm: The Swedish Council for Information on Alcohol and Other Drugs

[B8] Heron M Hoyert DL Murphy SL Xu J Kochanek KD Tejada-Vera B Deaths: final data for 2006 National vital statistics report: from the Centers for Disease Control and Prevention, National Center for Health Statistics, National Vital Statistics System 2009 57 1 134 19788058

[B9] Bonomo YA Bowes G Coffey C Carlin JB Patton GC Teenage drinking and the onset of alcohol dependence: a cohort study over seven years Addiction 2004 99 1520 1528 10.1111/j.1360-0443.2004.00846.x 15585043

[B10] Grant BF Stinson FS Harford TC Age at onset of alcohol use and DSM-IV alcohol abuse and dependence: a 12-year follow-up J Subst Abuse 2001 13 493 504 10.1016/S0899-3289(01)00096-7 11775078

[B11] Hingson RW Heeren T Winter MR Age at drinking onset and alcohol dependence. Age at onset, duration, and severity Arch Pediatr Adolesc Med 2006 160 739 746 10.1001/archpedi.160.7.739 16818840

[B12] Moore E Coffey C Carlin JB Alati R Patton GC Assessing alcohol guidelines in teenagers: results from a 10-year prospective study Aust N Z J Public Health 2009 33 154 159 10.1111/j.1753-6405.2009.00363.x 19413860

[B13] Bonomo YA Adolescent alcohol problems: whose responsibility is it anyway? Med J Aust 2005 183 430 432 16225452 10.5694/j.1326-5377.2005.tb07111.x

[B14] Honess T Seymour L Webster R The social contexts of underage drinking 2000 London: Home Office

[B15] Ryan SM Jorm AF Lubman DI Parenting factors associated with reduced adolescent alcohol use. A systematic review of longitudinal studies Aust NZ J Psychiatry 2010 44 774 783 10.1080/00048674.2010.501759 20815663

[B16] Jones J Hunter D Consensus methods for medical and health services research BMJ 1995 311 376 380 7640549 10.1136/bmj.311.7001.376 PMC2550437

[B17] Hart LM Jorm AF Kanowski L Kelly CM Langlands RL Mental health first aid for Indigenous Australians: using Delphi consensus studies to develop guidelines for culturally appropriate responses to mental health problems BMC Psychiatry 2009 9 47 10.1186/1471-244X-9-47 19646284 PMC2729076

[B18] Hart LM Jorm AF Paxton SJ Kelly CM Kitchener BA First aid for eating disorders Eating Disorders 2009 17 357 384 10.1080/10640260903210156

[B19] Kelly CM Jorm AF Kitchener BA Langlands RL Development of mental health first aid guidelines for suicidal ideation and behaviour: A Delphi study BMC Psychiatry 2008 8 17 10.1186/1471-244X-8-17 18366657 PMC2324091

[B20] Kelly CM Jorm AF Kitchener BA Langlands RL Development of mental health first aid guidelines for deliberate non-suicidal self-injury: A Delphi study BMC Psychiatry 2008 8 1 10 10.1186/1471-244X-8-1 18647420 PMC2518920

[B21] Kingston A Jorm A Kitchener B Hides L Kelly C Morgan A Hart L Lubman D Helping someone with problem drinking: Mental health first aid guidelines - a Delphi expert consensus study BMC Psychiatry 2009 9 79 10.1186/1471-244X-9-79 19968868 PMC2799400

[B22] Morgan AJ Jorm AF Self-help strategies that are helpful for sub-threshold depression: A Delphi consensus study J Affect Disord 2009 115 196 200 10.1016/j.jad.2008.08.004 18799220

