# Supplementary material for: Parenting strategies for reducing adolescent alcohol use: a Delphi consensus study
Source: BMC Public Health. 2011 Jan 6;11:13. doi: 10.1186/1471-2458-11-13 (PMC3022696; doi:10.1186/1471-2458-11-13)
Supplement: Additional file 1 — Table S1. Items endorsed and rejected from the 3 rounds of the study. [file 1471-2458-11-13-S1.DOC]

**Additional file 1**

*Table 1. Statements endorsed by the panel as important strategies for parents trying to prevent or reduce their adolescent’s alcohol use.*

| Round  endorsed | | Endorsed Statements | |
| --- | --- | --- | --- |
|  | ***Things parents should know*** | | |
| 1 | Parents should be aware that they can have a major influence on their adolescent child’s drinking, and can help prevent alcohol misuse. | | |
| 1 | Parents should be aware that they have more influence on their adolescent child’s attitudes and decisions about drinking before they begin drinking. | | |
| 1 | Parents should be aware that they play a major role in the decisions their adolescent child makes about drinking. | | |
| 1 | Parents should be aware that there are a range of strategies that they can employ to prevent their adolescent child from misusing alcohol. | | |
| 1 | In selecting strategies for reducing the risk of alcohol misuse for their adolescent, parents should choose approaches that are appropriate to the child’s maturity and personality. | | |
| 1 | Parents should be aware that experimentation and risk taking are a normal part of adolescence, and this may include drinking. | | |
| 1 | Parents should be aware that as a child approaches adolescence, friends and “fitting in” becomes extremely important. | | |
| 1 | Parents should be aware that alcohol is a drug. | | |
| 1 | Parents should be aware that alcohol is a depressant drug that has numerous effects on the body. | | |
| 1 | Parents should be aware of what National alcohol guidelines recommend to reduce health risks from drinking. | | |
| 1 | Parents should be aware of the potential alcohol-related harms specific to drinking during adolescence. | | |
| 1 | Parents should be aware that adolescents have less physical tolerance to the effects of alcohol. | | |
| 1 | Parents should be aware of the prevalence of adolescent drinking. | | |
| 1 | Parents should be aware of the additional risks associated with drinking during adolescence. | | |
| 1 | Parents should be aware that when adolescents drink, they are more likely to binge drink (drink a large amount in short space of time). | | |
| 1 | Parents should be aware that when adolescents drink, they often drink with the aim to get drunk. | | |
| 1 | Parents should be aware that whilst binge drinking is a common social behaviour amongst Australian adolescents, any episode of binge drinking is cause for concern. | | |
| 1 | Parents should be aware that a single episode of binge drinking does not mean an adolescent child has a drinking problem. | | |
| 1 | Parents should be aware of the reasons why adolescents drink. | | |
| 1 | Parents should be aware of the factors that may influence their adolescent child’s decision to drink. | | |
| 1 | Parents should be aware that adolescent drinking behaviour is influenced by what is considered normal within their cultural groups. | | |
| 1 | Parents should be aware that adolescents’ perceptions and attitudes about alcohol are influenced by how alcohol is marketed and portrayed in the media. | | |
| 2 | Parents should be aware that there is a good chance their adolescent child will have experimented with alcohol by the time they are 15 years of age. | |  |
| 2 | Parents should be aware that as their adolescent child gets older, the likelihood they will drink increases. | | |
| 2 | Parents should be aware of the additional risks associated with adolescent drinking unsupervised in public places, such as playgrounds and carparks. | | |
| 2 | Parents should be aware that many adolescents associate alcohol use with becoming an adult. | | |
| 2 | Parents should be aware that although it may sometimes seem like it, adolescents do not drink simply to disobey their parents. | | |
| 2 | Parents should be aware that adolescents are at an increased risk of alcohol misuse if they have behaviour problems. | | |
| 2 | Parents should be aware that adolescents are at an increased risk of alcohol misuse if they have emotional or psychological problems. | | |
| 2 | Parents should be aware that adolescents are at an increased risk of alcohol misuse if they do not feel connected to their family, school or community. | | |
| 2 | Parents should be aware of the risk factors that are associated with adolescent alcohol misuse. | | |
| 3 | Parents should be aware that if adolescents drink they often hide it from their parents. | | |
|  |  | | |
|  | ***Parental Modelling*** | | |
| 1 | Parents should be aware that they are important role models for their children, even during adolescence. | | |
| 1 | Parents should be aware that although there is a range risks associated with alcohol misuse, such as family history, parental attitude towards alcohol is a major influence upon an adolescent’s alcohol use. | | |
| 1 | Parents should be aware that the influence of parental modelling on their child’s attitude to alcohol begins at a very early age. | | |
| 1 | Parents should be aware that warning adolescents about the dangers of drinking will not be effective if they do not set a good example themselves. | | |
| 1 | Parents who drink should model responsible drinking by limiting their alcohol use, especially in front of their children. | | |
| 1 | Parents who drink should model responsible drinking by not getting drunk, especially in front of their children. | | |
| 1 | Parents who drink should model responsible drinking by establishing their own rules for drinking responsibly, and following them. | | |
| 1 | Parents who drink should model responsible drinking by providing food and non-alcoholic beverages if making alcohol available to guests. | | |
| 1 | Parents who drink should model responsible drinking by never drinking and driving. | | |
| 1 | Parents who drink should model responsible drinking by not letting other adults drive after they have been drinking. | | |
| 1 | Parents should model healthy ways to cope with stress without alcohol, such as exercise, listening to music, or talking things over. | | |
| 2 | Parents should be aware that what they themselves drink, how much, when and where they drink is a major influence on how their adolescent will drink in the future. | | |
| 2 | Parents who drink should model responsible drinking by explaining to their adolescent child the rules they follow in order to drink responsibly. | | |
| 2 | Parents who drink should model responsible drinking by sometimes declining the offer of alcohol. | | |
| 2 | Parents should not portray alcohol as a good way to deal with stress, such as by saying, “I’ve had a bad day, I need a drink!” | | |
| 3 | Parents should not convey the idea that alcohol is fun or glamorous to their children through stories about their own or other’s drinking. | | |
|  |  | | |
|  | ***Delaying initiation and introducing responsible drinking*** | | |
| 1 | Parents should be aware that early drinking (before the age of 15) is associated with an increase in later alcohol use. | | |
| 1 | Parents should be aware that early drinking (before the age of 15) is associated with an increase in later social problems associated with drinking. | | |
| 1 | Parents should be aware that the longer their adolescent delays alcohol use, the less likely they are to develop problems associated with alcohol. | | |
| 1 | Parents should never supply alcohol to their adolescent child’s friends. | | |
| 2 | Parents should be aware that early drinking (before the age of 15) is associated with later alcohol dependence. | | |
| 2 | Parents should be aware that early drinking (before the age of 15) is associated with later binge drinking. | | |
| 2 | Parents should be aware that they can teach responsible drinking to their adolescent without allowing the adolescent to drink. | | |
| 3 | Parents should aim to keep their adolescent child from experimenting with alcohol for as long as possible. | | |
| 3 | Parents should delay their adolescent child’s first alcoholic drink for as long as possible. | | |
| 3 | Parents should not give their adolescent child alcohol while they are under the age of 15. | | |
| 3 | Parents should tell their adolescent that any alcohol stored in the family home is strictly off limits to the adolescent and their friends. | | |
| 3 | If their adolescent is over 15 and interested in alcohol, parents should explain to the adolescent why it is best to wait until they are older to start drinking alcohol. | | |
|  |  | | |
|  | ***Talking to adolescents about alcohol*** | | |
| 1 | Parents should be aware that talking to their adolescent child about the risks associated with alcohol can help reduce their risk of alcohol-related harms. | | |
| 1 | Before talking to their child about alcohol, parents should think about the main points they want to discuss with the child. | | |
| 1 | Before talking to their child about alcohol, parents should think about questions their child may ask, and how they would respond to them. | | |
| 1 | Before talking to their child about alcohol, parents should be prepared to answer difficult questions. | | |
| 1 | Before talking to their child about alcohol, parents should clarify their own beliefs and attitudes regarding alcohol. For example is there a certain age when they think it is acceptable for an adolescent to try alcohol? | | |
| 1 | When talking to their child about alcohol, parents should choose a time when both the parent and the child are relaxed. | | |
| 1 | When talking to their child about alcohol, parents should tailor information and vocabulary to the child’s age and maturity. | | |
| 1 | When talking to their child about alcohol, parents should think about what they are conveying through their tone of voice, facial expressions and body language. | | |
| 1 | When talking to their child about alcohol, parents should remain calm. | | |
| 1 | When talking to their child about alcohol, parents should not respond with anger if they hear something they don’t like. | | |
| 1 | When talking to their child about alcohol, parents should listen when their child speaks, without interrupting. | | |
| 1 | When talking to their child about alcohol, parents should make it a conversation, not a lecture. | | |
| 1 | When talking to their child about alcohol, parents should show their child that they are listening by nodding, asking questions or repeating phrases back to the child. | | |
| 1 | When talking to their child about alcohol, parents should ask questions to make sure that the child understands what is being said. | | |
| 1 | When talking to their child about alcohol, parents should encourage the child to talk about anything that interests or concerns them about drinking. | | |
| 1 | When talking to their child about alcohol, parents should ask the child what they think about alcohol. | | |
| 1 | When talking to their child about alcohol, parents should ask their child why they think kids drink, and listen carefully. | | |
| 1 | When talking to their child about alcohol, parents should admit if they don’t know an answer, and look for it using a reputable source, e.g. books, reputable websites, or a health professional. | | |
| 1 | Parents should continue to talk to their child about alcohol throughout their adolescence. | | |
| 1 | Even if their adolescent chooses not to drink, parents should still discuss alcohol with them. | | |
| 1 | When talking to their adolescent child about alcohol, parents should tell them the facts about alcohol and its harms. | | |
| 1 | When talking to their adolescent child about alcohol, parents should talk about the positive as well as the negative effects of alcohol. | | |
| 1 | When talking to their adolescent child about alcohol, parents should avoid scare tactics and exaggerating its negative effects. | | |
| 1 | When talking to their adolescent child about alcohol, parents should address any myths or misinformation the child may have about alcohol. | | |
| 1 | When talking to their adolescent child about alcohol, parents should encourage them to question the assumption that most adolescents drink, and help them realise that many of their peers are not drinking. | | |
| 1 | When talking to their adolescent child about alcohol, parents should highlight and discuss myths or glamourisation of alcohol portrayed in the media. | | |
| 1 | When talking to their adolescent child about alcohol, parents should explain to them that their brain is still developing, and therefore more vulnerable to harm caused by alcohol. | | |
| 1 | When talking to their adolescent child about alcohol, parents should let them know the health benefits of choosing not to drink. | | |
| 1 | When talking to their adolescent child about alcohol, parents should teach them that different types of alcoholic drinks contain different amounts of alcohol. | | |
| 1 | When talking to their adolescent child about alcohol, parents should teach them that the effects of alcohol vary between individuals, depending upon the amount of alcohol, the person and the context. | | |
| 1 | When talking to their adolescent about alcohol, parents should discuss the added risks of using alcohol with other drugs. | | |
| 1 | When talking to their adolescent about alcohol, parents should tell them not to participate in potentially risky activities, such as skateboarding, swimming, or riding a bike, after they have consumed alcohol. | | |
| 1 | When talking to their adolescent about alcohol, parents should ensure they know the laws relating to underage alcohol consumption, drunkenness and drink driving. | | |
| 1 | Parents should be aware of how alcohol is addressed in their adolescent’s school curriculum. | | |
| 2 | Before talking to their child about alcohol parents should make sure that are knowledgeable about alcohol and its effects. | | |
| 2 | Before talking to their child about alcohol parents should evaluate their own behaviours and attitudes regarding alcohol. | | |
| 2 | When talking to their child about alcohol, parents should be clear, direct and specific. | | |
| 2 | When talking to their child about alcohol, parents should resist bringing up the child’s past mistakes. | | |
| 2 | When talking to their child about alcohol, parents should be prepared to listen without expressing judgment. | | |
| 2 | When talking to their adolescent child about alcohol, parents should address the myth that most adolescents get drunk. | | |
| 2 | When talking to their adolescent child about alcohol, parents should emphasise the short-term harms associated with alcohol, as these generally have a greater impact on adolescents. | | |
| 2 | When talking to their adolescent child about alcohol, parents should explain to them that alcohol may cause them to do something embarrassing that might damage their self-esteem and friendships. | | |
| 2 | When talking to their adolescent child about alcohol, parents should discuss with them how, if they do drink, they should do so in moderation. | | |
| 2 | When talking to their adolescent child about alcohol, parents should discuss with them how risks associated with alcohol can be minimised. | | |
| 2 | When talking to their adolescent child about alcohol, parents should discuss with them how, if they choose to drink when they are an adult, they should do so responsibly. | | |
| 2 | When communicating with their adolescent about alcohol and its harms, parents should think about how they best relate to the adolescent, and use methods that have worked well within that relationship. | | |
| 2 | Parents should not present a permissive approach to alcohol, as this can increase the likelihood of alcohol misuse by their adolescent child. | | |
| 2 | Parents should discuss with their adolescent their expectations of the adolescent regarding alcohol consumption. | | |
| 2 | Parents should discuss with their adolescent their expectations of the adolescent's alcohol use in specific contexts, for example at family celebrations or "Schoolies Week". | | |
| 3 | When talking to their adolescent child about alcohol, parents should give them valid reasons why they should not drink. | | |
| 3 | When talking to their adolescent child about alcohol, parents should discuss with them how the best way for them to avoid harms associated with alcohol is to not drink at all before the age of 15. | | |
|  |  | | |
|  | ***General discipline & rules about alcohol*** | | |
| 1 | When formulating a disciplinary approach for their adolescent, parents should be aware that clear general rules not specific to alcohol are important in protecting their adolescent child from alcohol misuse. | | |
| 1 | When formulating a disciplinary approach for their adolescent, parents should support the adolescent’s growing independence, whilst setting appropriate limits. | | |
| 1 | When formulating a disciplinary approach for their adolescent, parents should use positive reinforcement. | | |
| 1 | When establishing family rules parents should tell the child exactly what the rules are, and ensure they understand them. | | |
| 1 | When establishing family rules parents should be prepared to negotiate with their adolescent child on rules regarding minor matters. | | |
| 1 | When establishing family rules parents should ensure the child understands that those rules are to be maintained when the child is away from the family home. | | |
| 1 | Parents should maintain family rules by reviewing them as their adolescent shows more maturity and responsibility. | | |
| 1 | Parents should maintain family rules by supporting each other regarding family rules and presenting a united front. | | |
| 1 | In establishing family rules regarding alcohol, parents should ensure the adolescent knows that these rules are a protective measure, and not just a restriction on their freedom. | | |
| 1 | Parents should establish rules for whilst the adolescent is at home unsupervised. | | |
| 1 | Parents should establish and enforce a rule that their adolescent is to obtain their parents’ permission before holding a party or gathering in the family home. | | |
| 1 | Parents should establish and enforce a rule that their adolescent is not to get into a car driven by someone who has been drinking. | | |
| 1 | Parents should establish and enforce a rule that the adolescent must never drink alcohol and drive. | | |
| 1 | When establishing consequences for when family rules are broken, parents should ensure that they are realistic. | | |
| 1 | When establishing consequences for when family rules are broken, parents should make them very clear to their adolescent child. | | |
| 1 | When establishing consequences for when family rules are broken, parents should involve the child in their development. | | |
| 1 | When enforcing established consequences for when family rules are broken parents should enforce them consistently every time that family rules are broken. | | |
| 1 | When enforcing established consequences for when family rules are broken parents should explain to the child calmly why the behaviour results in that consequence. | | |
| 1 | When enforcing established consequences for when family rules are broken parents should make sure that their adolescent child knows that they are loved. | | |
| 2 | Parents should be aware that family rules are important in protecting their adolescent from alcohol misuse. | | |
| 2 | When establishing family rules parents should involve the adolescent in their development. | | |
| 2 | When establishing family rules parents should not change the family rules or consequences without discussing it with the adolescent. | | |
| 2 | When establishing family rules parents should let the adolescent know that they expect them to make wise choices based on family rules. | | |
| 2 | Parents should have family rules established before the adolescent is exposed to situations involving alcohol. | | |
| 2 | Parents should seek help with parenting if their adolescent continues to break alcohol-specific rules. | | |
| 3 | When establishing consequences for when rules are broken, parents should make sure they are harsh enough to be a deterrent, whilst ensuring they will not damage the parent-adolescent relationship should they be imposed. | | |
|  |  | | |
|  | ***Supervision & monitoring*** | | |
| 1 | Parents should be aware that adolescents are more likely to misuse alcohol when adults are not around. | | |
| 1 | Parents should be aware that parental monitoring reduces the likelihood of their adolescent misusing alcohol. | | |
| 1 | Parents should monitor their adolescent by asking them where they will be when they are unsupervised. | | |
| 1 | Parents should monitor their adolescent by asking them what they will be doing when they are unsupervised. | | |
| 1 | Parents should monitor their adolescent by asking them who they will be with when they are unsupervised. | | |
| 1 | Parents should monitor their adolescent by establishing a curfew for the adolescent when they go out at night unsupervised. | | |
| 1 | Parents should monitor their adolescent by knowing what time to expect the adolescent home when they go out. | | |
| 1 | Parents should monitor their adolescent by asking the adolescent to contact the parent to let them know if their plans change when they are out unsupervised. | | |
| 1 | Parents should monitor their adolescent by ensuring that the adolescent has a means by which they can contact the parent if their plans change when they are out unsupervised. | | |
| 1 | Parents should monitor their adolescent by making arrangements with their adolescent to get home safely before they go out at night unsupervised. | | |
| 1 | Parents should make sure they know where their adolescent is when they are going out, especially if they or others may be drinking. | | |
| 1 | Parents should be aware that although monitoring their adolescent’s activities reduces the likelihood of them misusing alcohol, an authoritarian approach, and harsh discipline may cause the adolescent to rebel by misusing alcohol. | | |
| 1 | Parents should balance their need to monitor with the adolescent’s need for privacy. | | |
| 1 | Parents should be aware that as their adolescent matures, the parents approach to monitoring should be adjusted to encourage the child’s growing independence. | | |
| 1 | Parents should tell their adolescent that they are monitoring the child’s activities not because they are nosey, but because they care about the child’s safety. | | |
| 2 | Parents should monitor their adolescent by checking in with the host parents when their adolescent is out at a party or a sleep over. | | |
| 2 | Parents should be aware that most adolescents appreciate their parents monitoring their activities, and see it as proof of their parent’s concern for their well-being. | | |
| 3 | Before giving their adolescents money, parents should discuss with them how much they need for particular activities, and how it will be spent. | | |
|  |  | | |
|  | ***Relationship quality*** | | |
| 1 | Parents should be aware that a close, supportive relationship with their adolescent does not guarantee that their adolescent will not misuse alcohol. | | |
| 1 | Parents should establish and maintain a close, supportive relationship with their adolescent child by working to create open communication between themselves and the adolescent. | | |
| 1 | Parents should establish and maintain a close, supportive relationship with their adolescent child by showing an interest and being involved in the adolescent’s life. | | |
| 1 | Parents should establish and maintain a close, supportive relationship with their adolescent child by supporting their adolescent in pursuing their interests, and in dealing with problems. | | |
| 1 | Parents should establish and maintain a close, supportive relationship with their adolescent child by cultivating their adolescent’s trust by being consistent in following through on promises and enforcing rules. | | |
| 1 | Parents should establish and maintain a close, supportive relationship with their adolescent child by regularly demonstrating to the adolescent that they care about them. | | |
| 1 | Parents should establish and maintain a close, supportive relationship with their adolescent child by regularly telling the adolescent that they love them. | | |
| 1 | Parents should help their adolescent to feel good about themselves. | | |
| 1 | Parents should praise their adolescent for their good behaviour. | | |
| 1 | Parents should praise their adolescent for their efforts as well as their achievements. | | |
| 2 | Parents should be aware that a close, supportive relationship with their adolescent reduces the likelihood of their adolescent misusing alcohol. | | |
| 2 | Parents should be aware that a close, supportive relationship with their adolescent will influence how effective their efforts are in protecting their adolescent from alcohol misuse. | | |
| 2 | Parents should be aware that a close, supportive relationship with their adolescent will encourage the adolescent to seek help from the parent should they face an issue relating to alcohol. | | |
| 2 | Parents should be aware that it is hard to be a parent, and not hesitate to seek advice if having difficulties with parenting. | | |
|  |  | | |
|  | ***Family conflict*** | | |
| 1 | Parents should ensure that their positive comments outweigh their negative comments in their interactions with their adolescent. | | |
| 1 | Parents should not tease their adolescent in a way that could be perceive as hurtful. | | |
| 1 | Parents should obtain advice from professional sources if they feel it would benefit their relationship with their adolescent. | | |
| 2 | Parents should avoid actions and statements that their adolescent is likely to interpret as rejection. | | |
|  |  | | |
|  | ***Parental support*** | | |
| 1 | Parents should monitor their adolescent child for signs of high stress. | | |
| 1 | Parents should be aware that adolescents experiencing mental health problems such as anxiety or depression are at an increased risk of alcohol misuse. | | |
| 1 | Parents should monitor their adolescent child for signs of mental health problems. | | |
| 1 | Parents should provide their adolescent with additional support during periods of high stress, e.g. starting high school, changing schools, or major exams. | | |
| 1 | Parents should encourage their adolescent to use healthy approaches to dealing with stress, like exercise, music or talking over problems. | | |
| 1 | Parents should help their adolescent to cope with disappointment. | | |
| 1 | Parents should encourage their adolescent to accept the consequences of their choices. | | |
| 1 | Parents should give their adolescent a chance to solve their own problems, as this will help them build self-esteem. | | |
| 1 | Parents should be aware that adolescents are less likely to misuse alcohol if their parents are involved in their lives. | | |
| 1 | Parents should be aware that spending time with their adolescent facilitates communication between the parent and the adolescent. | | |
|  |  | | |
|  | ***Parental involvement*** | | |
| 1 | Parents can be involved with their adolescent by regularly spending one-on-one time with the adolescent, giving them their undivided attention. | | |
| 1 | Parents can be involved with their adolescent by engaging in activities together as a family on a regular basis. | | |
| 1 | Parents can be involved with their adolescent by trying to eat dinner together as a family to facilitate communication with their children. | | |
| 1 | Parents can be involved with their adolescent by including their adolescent’s friends in family activities. | | |
| 2 | Parents can be involved with their adolescent by establishing a regular weekly routine for doing something special with the adolescent. | | |
| 2 | Parents can be involved with their adolescent by finding ways for their adolescent to be involved in family life, such as doing chores or caring for younger brothers or sisters. | | |
|  |  | | |
|  |  | | |
|  | ***General communication*** | | |
| 1 | Parents should be aware that good communication between themselves and their adolescent can reduce the risk of their adolescent misusing alcohol. | | |
| 1 | Parents should encourage communication with their adolescent by asking the adolescent about topics that interest them, and listening to them when they talk. | | |
| 1 | Parents should encourage communication with their adolescent by encouraging the adolescent to express their opinions during everyday conversations. | | |
| 1 | Parents should encourage communication with their adolescent by encouraging the adolescent to discuss their problems and concerns with the parent. | | |
| 1 | Parents should encourage communication with their adolescent by avoiding questions that have simple “yes” or “no” answers, and instead asking open-ended questions when talking to the adolescent. | | |
| 1 | Parents should encourage communication with their adolescent by talking to the adolescent regularly one-on-one. | | |
| 1 | Parents should encourage communication with their adolescent by not responding with anger if they hear something they don’t like when talking to their adolescent. | | |
| 1 | Parents should encourage communication with their adolescent by avoiding interrogating the adolescent, as this may cause the adolescent to be less open. | | |
| 1 | Parents should encourage communication with their adolescent by not making all one-on-one time with the adolescent a time for deep discussion, as the child may begin to avoid these situations. | | |
| 2 | Parents should encourage communication with their adolescent by trying to make themselves available to listen to their adolescent whenever they need it. | | |
| 2 | If parents need help communicating with their adolescent they should get advice from someone with expert knowledge about parenting. | | |
| 3 | Parents should be aware that their adolescent will respect them more if they admit they don't have all the answers. | | |
| 3 | When talking with their adolescent, parents should be aware that adolescents are often reluctant to talk about sensitive issues such as alcohol. | | |
|  |  | | |
|  | ***Peer influence*** | | |
| 1 | Parents should be aware that their adolescent’s friends are a major influence on the adolescent’s decisions about alcohol use. | | |
| 1 | Parents should be aware that if their adolescent’s friends use alcohol, their adolescent is more likely to use alcohol. | | |
| 1 | Parents should be aware that despite the growing influence of peers on their adolescent’s alcohol use, parents are still able to exert a positive influence on their adolescent’s alcohol use. | | |
| 1 | Parents should be aware that their adolescent may find themselves in situations where it is difficult for them to say no to alcohol, because of peer pressure. | | |
| 1 | Parents should be aware that peer pressure can be a positive influence. | | |
| 1 | Parents should tell their adolescent that the decision as to whether or not to drink is theirs, and not their friends’. | | |
| 1 | Parents should encourage their adolescent to support others who experience pressure to use alcohol from their peers. | | |
| 1 | Parents should get to know their adolescent's friends. | | |
| 1 | Parents should get to know their adolescent’s friends by talking and interacting with them. | | |
| 1 | Parents should encourage their adolescent to invite their friends over when the parent is at home. | | |
| 1 | Parents should encourage their adolescent to invite their friends over as this allows the parent to get to know the friends better, as well as learn about their child’s activities. | | |
| 1 | Parents should be aware that their adolescent's friends' families may have different values and attitudes regarding alcohol to their own, and this may cause some difficulty in maintaining rules regarding alcohol for their adolescent. | | |
| 1 | Parents should talk to their adolescent about qualities that really count in a friend, such as being kind and trustworthy, rather than popular and “cool”. | | |
| 2 | Parents should be aware that negative influences exerted by their adolescent’s friends regarding alcohol are greater when the relationship between the parent and the adolescent is of a poorer quality. | | |
| 2 | Parents should build a support network with other parents. | | |
| 3 | Parents should be aware that obtaining acceptance from peers can be extremely difficult for their child during adolescence. | | |
|  |  | | |
|  | ***Preparation for situations involving alcohol*** | | |
| 1 | Parents should discuss with their adolescent child how to deal with peer pressure to drink. | | |
| 1 | Parents should discuss with their adolescent situations they may be faced with where they are pressured to drink to ensure they are sufficiently prepared for handling these situations. | | |
| 1 | Parents should discuss with their adolescent ways to deal with peer pressure to drink. | | |
| 1 | Parents should discuss with their adolescent situations they may be faced with where other people are misusing alcohol. | | |
| 1 | When talking to their adolescent about situations involving alcohol, parents should help the adolescent to develop strategies for handling these situations. | | |
| 1 | Parents should help their adolescent develop strategies for removing themselves from situations involving alcohol misuse. | | |
| 1 | Parents should make it clear to the adolescent the ways in which they will support them in dealing with situations involving alcohol, such as offering to pick them up. | | |
| 1 | Parents should tell their adolescent to call them if ever faced with a situation involving alcohol and assure them they will pick them up, whatever the circumstances. | | |
| 1 | Parents should discuss with the adolescent ways to minimise any potential embarrassment that may be associated with getting picked up by their parents from situations involving alcohol. | | |
| 1 | Parents should warn their adolescent about the dangers of getting into a car driven by someone who has been drinking. | | |
| 1 | Parents should establish a plan for when the adolescent is faced with a drink driver, such as agreeing to pay for a taxi, or picking them up. | | |
| 1 | Parents should let the adolescent know that there will be no negative repercussions for calling the parent for a lift when faced with a situation involving a drink driver. | | |
| 1 | Parents should give their adolescent positive feedback when they handle a situation involving alcohol well. | | |
| 2 | When talking to their adolescent about situations involving alcohol, parents should focus on specific situations that the adolescent may encounter. | | |
| 2 | Parents should help their adolescent to develop refusal techniques for offers of alcohol before they are faced with situations where they are offered alcohol. | | |
| 2 | Parents should talk to their adolescent about the dangers of drink "spiking", for example how someone can deliberately put a dangerous amount of alcohol in a drink with the aim of intoxicating another person, and how even non-alcoholic drinks can be spiked with alcohol. | | |
| 2 | Parents should talk to their adolescent about the dangers of drink "spiking" and how they can protect themselves. | | |
|  |  | | |
|  | ***Activities and community action*** | | |
| 1 | Parents should discuss with their adolescent how they might support the child’s interests and activities. | | |
| 1 | Parents should encourage their adolescent to find ways to have fun without alcohol. | | |
| 2 | Parents should encourage their adolescent to participate in supervised groups, clubs, and events that are fun, challenging, and alcohol free. | | |
|  |  | | |
|  | ***Parties*** | | |
| 1 | When allowing their adolescent to attend a party, parents should get the name and number of the responsible adult who will be supervising the party. | | |
| 1 | Before allowing their adolescent to attend a party, parents should contact the party host to confirm whether it will be adequately supervised. For example; if a responsible adult will be there, if alcohol will be allowed, if alcohol consumption will be regulated, if the party is invite only, and what time it will finish. | | |
| 1 | Parents should talk to the person driving when their adolescent is going out and confirm with them that they will not be drinking. | | |
| 1 | Parents should discuss with their adolescent strategies for minimising harm associated with alcohol when they are out drinking with friends, such as sticking with their friends, not walking off alone, and ensuring that others know where they are. | | |
| 1 | Parents should be aware of the range of settings in which unsupervised adolescent drinking occurs. | | |
| 1 | Parents should be aware of the range of sources that adolescents can obtain their alcohol from for unsupervised drinking. | | |
| 1 | Parents should be aware of the laws regarding providing alcohol to their child whilst they are underage. | | |
| 1 | When hosting an adolescent party, parents should ensure that there is adequate adult supervision. | | |
| 1 | When hosting an adolescent party, parents should work with their adolescent to plan age appropriate activities to take the focus off drinking at the party. | | |
| 1 | When hosting an adolescent party, parents should be aware of the legal responsibilities of hosting an adolescent party where alcohol is consumed. | | |
| 1 | When hosting an adolescent party, parents should make a clear decision as to whether or not alcohol will be allowed at the party. | | |
| 1 | When hosting an adolescent party, parents should communicate their rules regarding alcohol to all guests attending the party. | | |
| 1 | When hosting an adolescent party, parents should know strategies for de-escalating potential violence. | | |
| 1 | When hosting an adolescent party, parents should consider how they will prevent or manage gatecrashers. | | |
| 1 | If they have decided not to allow alcohol at an adolescent party, parents should provide their adolescent with reasons as to why they will not allow alcohol at the party. | | |
| 2 | Parents should not allow their adolescent to attend an adolescent party that is not adequately supervised. | | |
| 2 | When their adolescent is attending an adolescent party, parents should make sure they have a safe ride with a responsible adult to and from the party. | | |
| 2 | Parents should be aware that providing their adolescent with alcohol for parties and gatherings increases the likelihood that the adolescent will misuse alcohol. | | |
| 2 | Parents should be aware that providing their adolescent with alcohol for adolescent parties and gatherings increases the likelihood that the adolescent will misuse alcohol. | | |
| 2 | When hosting an adolescent party, parent should consider advice provided by reputable "partysafe" websites such as those produced by state police and the ADF. | | |
| 2 | Parents should not allow alcohol consumption at any parties for adolescents under the age of 15. | | |
| 2 | When allowing some alcohol consumption at an adolescent party they are hosting, parents should implement strategies to prevent alcohol misuse by guests. | | |
| 2 | When allowing some alcohol consumption at an adolescent party they are hosting, parents should .implement strategies to prevent alcohol misuse by guests recommended by reputable "partysafe" websites such as those produced by state police and the ADF. | | |
| 2 | When allowing some alcohol consumption at an adolescent party they are hosting, parents should discuss the rules regarding alcohol with their adolescent before inviting people to the party. | | |
| 2 | When allowing some alcohol consumption at an adolescent party they are hosting, parents should ensure that parents of guests are informed that there will be alcohol at the party, as well as what restrictions will be in place to prevent alcohol misuse. | | |
| 2 | When allowing some alcohol consumption at an adolescent party they are hosting, parents should make it clear to their adolescent and guests that drunkenness will not be tolerated. | | |
| 2 | When allowing some alcohol consumption at an adolescent party they are hosting, parents should confiscate alcohol if necessary. | | |
| 2 | When allowing some alcohol consumption at an adolescent party they are hosting, parents should ensure they have a strategy prepared for if someone drinks too much. | | |
| 2 | When allowing some alcohol consumption at an adolescent party they are hosting, parents should ensure that a responsible adult who knows first aid is present at the party. | | |
| 2 | When allowing some alcohol consumption at an adolescent party they are hosting, parents should not allow guests who have been drinking to drive home. | | |
| 2 | When allowing some alcohol consumption at an adolescent party they are hosting, parents should make sure that guests have a safe ride to and from the party with a responsible adult. | | |
| 2 | When allowing some alcohol consumption at an adolescent party they are hosting, parents should limit their own alcohol consumption. | | |
| 3 | When their adolescent is attending a party, parents should make sure they have a safe ride with a responsible adult to and from the party. | | |
|  |  | | |
|  | ***When an adolescent has been drinking without permission*** | | |
| 1 | Parents should be aware that despite their best efforts they may not be able to prevent their adolescent from drinking. | | |
| 1 | Parents should be aware that despite their best efforts they may not be able to prevent their adolescent from misusing alcohol. | | |
| 1 | Parents should know the signs that indicate that their adolescent may be misusing alcohol. | | |
| 1 | If parents are concerned that their adolescent may be misusing alcohol they should talk to the adolescent directly about their concerns. | | |
| 1 | If their adolescent comes home drunk, parents should wait until the adolescent is sober before talking to them about their behaviour. | | |
| 1 | Parents should know how to respond if their adolescent has a medical emergency due to intoxication. | | |
| 1 | If parents are concerned that their adolescent may be misusing alcohol they should try to stay calm. | | |
| 1 | If parents are concerned that their adolescent may be misusing alcohol they should allow the adolescent to tell their side of the story. | | |
| 1 | If parents are concerned that their adolescent may be misusing alcohol they should not lecture the adolescent. | | |
| 1 | If parents are concerned that their adolescent may be misusing alcohol they should tell the adolescent what concerns them about the child’s alcohol use, such as that they broke the rules, and/or that they put their health and safety at risk. | | |
| 1 | If parents are concerned that their adolescent may be misusing alcohol they should take care that they communicate to the adolescent that they disapprove of the behaviour, not the adolescent themselves. | | |
| 1 | If parents are concerned that their adolescent may be misusing alcohol they should use ‘I’ statements, such as ‘I feel very upset about you breaking the family rules’ rather than ‘you are a deceitful, untrustworthy child’. | | |
| 1 | Following an incident where their adolescent child has misused alcohol, parents should be aware that one or a small number of incidents of alcohol misuse in adolescence does not mean the child will develop an alcohol problem. | | |
| 1 | Following an incident where their adolescent child has misused alcohol, parents should use dealing with the adolescent’s alcohol misuse as an opportunity to maintain or improve communication between the parent and the adolescent. | | |
| 1 | Following an incident where their adolescent child has misused alcohol, parents should learn as much as they can about the adolescent’s understanding and beliefs about alcohol, and about how drinking makes them feel. | | |
| 2 | Although they should be concerned if their adolescent has been drinking, parents should bear in mind that many adolescents drink, and few develop alcohol problems. | | |
| 2 | If parents find evidence of their adolescent breaking family rules relating to alcohol they should not ignore it. | | |
| 2 | If they are unsure as to how to approach their adolescent about their alcohol misuse, parents should consider enlisting the help of someone knowledgeable, such as a family doctor or a counsellor. | | |

Table 2. Items rejected by the panel for inclusion as a parenting strategy to reduce or prevent adolescent alcohol use

|  | Round  Rejected | | | Rejected Statements |  | |
| --- | --- | --- | --- | --- | --- | --- |
|  | | | ***Things parents should know*** | | |  |
|  | | 1 | Parents should be aware that adolescents tend to drink more often as they get older. | | | |
|  | | 1 | Parents should be aware that most adolescents do not drink unsupervised on a regular basis. | | | |
|  | | 1 | Parents should be aware adolescents who binge drink are more likely to develop alcohol-related problems later in life. | | | |
|  | | 1 | Parents should be aware the majority of adolescents who binge drink do not develop alcohol-related problems later in life. | | | |
|  | | 1 | Parents should consider any drinking by their adolescent cause for concern whilst they are under the age of 18. | | | |
|  | | 3 | In selecting strategies for reducing the risk of alcohol misuse for their adolescent, parents should choose approaches that are consistent with their overall parenting approach. | | | |
|  | | 3 | Parents should be able to differentiate between normal adolescent behaviours and behaviours that indicate that their adolescent is misusing alcohol. | | | |
|  | | 3 | Parents should consider any drinking by their adolescent cause for concern whilst they are under the age of 15. | | | |
|  | | 3 | Parents should be aware that parents often underestimate their adolescent’s level of drinking. | | | |
|  | | 3 | Parents should be aware that many adolescents first experiment with alcohol out of curiosity. | | | |
|  | | 3 | Parents should be aware that adolescents are at an increased risk of alcohol misuse if they have a strong desire for new experiences and sensations. | | | |
|  | | 3 | Parents should be aware that adolescents are at an increased risk of alcohol misuse if they are impulsive. | | | |
|  | | 3 | Parents should be aware that adolescents are at an increased risk of alcohol misuse if they have a family member who has experienced problems with alcohol. | | | |
|  | | 3 | Parents should be aware that adolescents are at an increased risk of alcohol misuse if they have mental health problems. | | | |
|  | | |  | | |  |
|  | | | ***Parental Modelling*** | | |  |
|  | | 1 | Parents should not drink in front of their children. | | | |
|  | | 1 | If parents do not drink, they should explain to their adolescent child their reasons for not drinking, whether they are religious, health related, or due to a family history of alcohol problems. | | | |
|  | | 1 | Parents who drink should model responsible drinking by making some family gatherings alcohol-free. | | | |
|  | | 1 | Parents should never ask their children to bring them an alcoholic drink, or to prepare them an alcoholic beverage. | | | |
|  | | 1 | Parents should be cautious about what they say about alcohol in front of their children. | | | |
|  | | 1 | Parents should not make alcohol an important part of celebrations. | | | |
|  | | 1 | Parents should identify important community figures or celebrities who are good role models for responsible drinking to their children. | | | |
|  | | 3 | Parents should be aware that adolescents are highly likely to apply their parents' point of view to their own friends and social situations. | | | |
|  | | 3 | Parents who drink should model responsible drinking by sometimes not drinking at social events. | | | |
|  | | |  | | |  |
|  | | | ***Delaying Initiation & Introducing Responsible Drinking*** | | |  |
|  | | 1 | Parents should not give their adolescent child alcohol when they are under the age of 18. | | | |
|  | | 1 | Parents should be aware that by allowing their adolescent to drink responsibly in the family home they are projecting a permissive attitude that increases the likelihood of their adolescent misusing alcohol. | | | |
|  | | 1 | Parents should be aware that by allowing their adolescent to drink responsibly in the family home they reduce the likelihood of the adolescent experimenting with alcohol in an unsupervised setting. | | | |
|  | | 1 | Parents should be aware that introducing their adolescent to alcohol in the family home reduces the novelty and appeal of alcohol to the adolescent, making them less likely to drink unsupervised. | | | |
|  | | 1 | Parents should be aware that if their adolescent chooses to drink, supervising their drinking will reduce the risk of harms associated with drinking. | | | |
|  | | 1 | Parents should be aware that early exposure to alcohol, as is done in some other countries, may not be effective in preventing alcohol misuse within the Australian context. | | | |
|  | | 1 | If their adolescent is over 15 and interested in alcohol, parents should introduce the child to alcohol by allowing the child to drink at home responsibly. | | | |
|  | | 1 | If their adolescent is over 15 and interested in alcohol, parents should introduce alcohol to the child gradually, in small amounts. | | | |
|  | | 1 | If their adolescent is over 15 and interested in alcohol, parents should introduce alcohol to the child by allowing them to drink only when the parent is drinking. | | | |
|  | | 1 | If their adolescent is over 15 and interested in alcohol, parents should allow the child to drink occasionally at mealtimes. | | | |
|  | | 1 | If their adolescent is over 15 and interested in alcohol, parents should never allow their adolescent to drink. | | | |
|  | | 1 | Parents should not keep any alcohol within the family home. | | | |
|  | | 1 | Parents should store alcohol in the family home in a place that cannot be accessed by children. | | | |
|  | | 1 | Parents should keep track of any alcohol kept within the family home. | | | |
|  | | 1 | Parents should never supply alcohol to their adolescent child’s friends without the permission of their parents’. | | | |
|  | | 3 | If their adolescent is over 15 and going to drink, parents should be aware that it is better that they do so under parental supervision. | | | |
|  | | |  | | |  |
|  | | | ***Talking to adolescents about alcohol*** | | |  |
|  | | 1 | Parents should be aware that for most adolescents their parents are the main source of information about alcohol. | | | |
|  | | 1 | Parents should be aware that most adolescents prefer their parents to be their main source of information about alcohol. | | | |
|  | | 1 | Parents should start talking to their child about alcohol when they are young. | | | |
|  | | 1 | Parents should broach the topic of alcohol with their child by using prompts from television, advertising or newspapers. | | | |
|  | | 1 | Parents should broach the topic of alcohol with their child by using any blocks of time spent with the child, such driving to school or over family meals. | | | |
|  | | 1 | Parents should broach the topic of alcohol with their child by taking advantage of times when their adolescent child wants something from the parent. | | | |
|  | | 1 | Parents should broach the topic of alcohol with their child when people present are drinking. | | | |
|  | | 1 | Parents should broach the topic of alcohol with their child when the parent is drinking themselves. | | | |
|  | | 1 | Parents should not be drinking or affected by alcohol when discussing alcohol with their child. | | | |
|  | | 1 | When talking to their child about alcohol, parents should minimise any distractions, such as television or telephones. | | | |
|  | | 1 | When talking to their child about alcohol, parents should talk to each child individually if they have more than one child. | | | |
|  | | 1 | When talking to their child about alcohol, parents should avoid making their child’s behaviour the centre of the discussion. | | | |
|  | | 1 | Parents should be aware that expressing their disapproval of underage drinking can reduce the likelihood of their adolescent drinking underage. | | | |
|  | | 1 | Parents should not present a permissive approach to alcohol, as this can increase the likelihood of alcohol misuse by their adolescent. | | | |
|  | | 1 | When talking to their adolescent child about alcohol, parents should discuss the “Australian guidelines to reduce the health risks from drinking alcohol” | | | |
|  | | 1 | When talking to their adolescent child about alcohol, parents should be open and honest about their own experiences. | | | |
|  | | 1 | When talking to their adolescent about alcohol, parents should tell them that the best way for them to avoid harms associated with alcohol is to not drink at all before the age of 18. | | | |
|  | | 1 | When talking to their adolescent about alcohol, parents should appeal to their self respect, for example by telling them they are too smart to risk their future by drinking. | | | |
|  | | 1 | When talking to their adolescent about alcohol, parents should emphasise the harm that drinking can cause to a person’s appearance, as adolescents often place great importance on their looks. | | | |
|  | | 1 | When talking to their adolescent about alcohol, parents should tell them that alcohol contains a lot of calories, and may cause weight gain. | | | |
|  | | 1 | When talking to their adolescent about alcohol, parents should let them know that they are more vulnerable to developing an alcohol problem if a family member has experienced a problem with alcohol. | | | |
|  | | 1 | When talking to their adolescent about alcohol, parents should let them know that they are more vulnerable to developing an alcohol problem if a family member has experienced a mental illness. | | | |
|  | | 1 | Parents should be aware that expressing their disapproval of underage drinking can reduce the likelihood of their adolescent drinking whilst underage. | | | |
|  | | 1 | Parents should explicitly tell to their adolescent child that they expect the child not to drink alcohol while they are under 18 years of age. | | | |
|  | | 1 | Parents should explicitly tell to their adolescent child any drinking by people under 18 years of age is unacceptable. | | | |
|  | | 1 | Parents should seek out and make use of local resources relating to prevention of adolescent alcohol misuse. | | | |
|  | | 2 | If parents need help discussing alcohol with their adolescent they should obtain advice from someone with expert knowledge about parenting. | | | |
|  | | 2 | When talking to their adolescent child about alcohol, parents should tell them that the best way for them to avoid harms associated with alcohol is to not drink at all before the age of 15. | | | |
|  | | 2 | When talking to their adolescent child about alcohol, parents should tell them that if they do drink they should do so in moderation. | | | |
|  | | 2 | When talking to their adolescent child about alcohol, parents should tell them how to minimise risks associated with drinking. | | | |
|  | | 2 | When talking to their adolescent child about alcohol, parents should tell them that, if they choose to drink when they are an adult, they should do so responsibly. | | | |
|  | | 2 | Parents should explicitly tell their adolescent that they expect the adolescent not to drink while they are under 15 years of age. | | | |
|  | | 2 | Parents should explicitly tell their adolescent exactly what they expect of the adolescent regarding alcohol consumption. | | | |
|  | | 3 | Parents should start talking to their child about alcohol before they start high school. | | | |
|  | | 3 | Parents should broach the topic of alcohol with their child by taking advantage of naturally arising opportunities, for example seeing or hearing about someone who has a drinking problem. | | | |
|  | | 3 | Parents should have numerous short conversations about alcohol with their child, rather than one long conversation. | | | |
|  | | 3 | When talking to their adolescent child about alcohol, parents should choose an environment that is as comfortable and non-threatening as possible, and where there is privacy for discussion. | | | |
|  | | 3 | When talking to their adolescent child about alcohol, parents should discuss why alcohol is especially bad for young people. | | | |
|  | | 3 | When talking to their adolescent child about alcohol, parents should provide them with tips for low risk drinking. | | | |
|  | | 3 | When talking to their adolescent child about alcohol, parents should let the adolescent know that they trust the adolescent to make the right decisions regarding alcohol. | | | |
|  | | 3 | If parents are concerned that alcohol is not being adequately covered by the school curriculum, they should supplement it at home. | | | |
|  | | 3 | Parents should consider talking to other parents to find out what parenting skills and strategies they find effective. | | | |
|  | | |  | | |  |
|  | | | ***General Discipline & Rules about Alcohol*** | | |  |
|  | | 1 | Parents should maintain family rules by writing out the most important ones and posting them where they can be seen clearly and often by family members. | | | |
|  | | 1 | Parents should maintain family rules by formalising their adolescent’s commitment to them with a family contract. | | | |
|  | | 1 | Parents should maintain family rules by reviewing them regularly as a family, for example on the child’s birthday, or New Year's day. | | | |
|  | | 1 | In establishing family rules regarding alcohol, parents should ensure that they reflect the parents' beliefs and values regarding alcohol. | | | |
|  | | 1 | Parents should establish rules regarding the amount and type of television and magazines the adolescent is allowed to view based on alcohol promotion. | | | |
|  | | 1 | Parents should establish rules about an appropriate time for when the adolescent is allowed to drink. | | | |
|  | | 1 | Parents should establish and enforce a rule that their adolescent is not to consume any alcohol while they are under 15. | | | |
|  | | 1 | Parents should establish and enforce a rule that their adolescent is not to consume any alcohol while they are under 18. | | | |
|  | | 1 | Parents should establish and enforce a rule that their adolescent is not to consume alcohol unsupervised by an adult. | | | |
|  | | 2 | Parents should establish rules about an appropriate age for when the adolescent is allowed to drink. | | | |
|  | | 2 | Parents should establish rules about the amount of alcohol the adolescent is allowed to consume when they drink, which corresponds with national guidelines for alcohol consumption. | | | |
|  | | 2 | Parents should ensure that their adolescent understands that their alcohol-specific rules are based upon national health guidelines. | | | |
|  | | 2 | Parents should discuss their expectations regarding alcohol with their adolescent, rather than setting alcohol-specific rules. | | | |
|  | | 2 | Parents should reinforce the alcohol-specific rules to their adolescent before each situation involving alcohol. | | | |
|  | | 2 | Parents should not apply different rules regarding alcohol based on gender. | | | |
|  | | 2 | Parents should discuss their alcohol-specific rules with the parents of their adolescent’s friends. | | | |
|  | | 2 | Parents should agree on a reward with the adolescent for adhereing to alcohol-specific rules. | | | |
|  | | 2 | Parents should get professional help for their adolescent if they continue to break alcohol-specific rules. | | | |
|  | |  |  | | | |
|  | | | ***Supervision & Monitoring*** | | |  |
|  | | 1 | Parents should establish and enforce a rule that their adolescent is not to entertain groups of friends when they are at home unsupervised. | | | |
|  | | 1 | Parents should establish and enforce a rule that their adolescent should not hold any parties or gatherings in the family home when the parents are not at home. | | | |
|  | | 1 | Parents should limit the amount of time their adolescent spends unsupervised by a responsible adult. | | | |
|  | | 1 | Parents should ensure that their adolescent is supervised by a responsible adult, not by an older sibling. | | | |
|  | | 1 | Parents should try to arrange to be at home when their adolescent gets home from school. | | | |
|  | | 1 | Parents should enlist the help of relatives, friends and other parents in organising supervision for their adolescent. | | | |
|  | | 1 | Parents should ensure their adolescent is supervised by a responsible adult when they are spending time with friends after school. | | | |
|  | | 1 | Parents should be aware that granting their child too much independence too early can lead to alcohol misuse. | | | |
|  | | 1 | Parents should monitor their adolescent by telling them what they are allowed to do, where they are allowed to go, and who they are allowed to spend time with when they are unsupervised. | | | |
|  | | 1 | Parents should monitor their adolescent by being awake and greeting the adolescent when they get home. | | | |
|  | | 1 | Parents should monitor their adolescent by being awake and greeting the adolescent when they get home, to confirm that the child has not been misusing alcohol. | | | |
|  | | 1 | Parents should monitor their adolescent by asking the adolescent to contact them regularly when they are out unsupervised. | | | |
|  | | 1 | Parents should monitor their adolescent by asking the adolescent contact them regularly whilst they are out unsupervised to ensure they are not misusing alcohol. | | | |
|  | | 1 | Parents should monitor their adolescent by checking in with the host parents when their adolescent is out at a party or a sleep over. | | | |
|  | | 1 | Parents should only give their adolescent the amount of money required for specific activities. | | | |
|  | | 1 | If their adolescent has a credit card, parents should monitor their spending with it for purchases of alcohol. | | | |
|  | | 2 | Parents should limit the amount of time adolescents under the age of 15 spend unsupervised by a responsible adult. | | | |
|  | | 2 | Parents should ensure that adolescents under the age of 15 are supervised by a responsible adult, and not by an older sibling. | | | |
|  | | 2 | If they are concerned their adolescent may be breaking alcohol-specific rules, parents should restrict the amount of time the adolescent is allowed out unsupervised. | | | |
|  | | 3 | Parents should provide their adolescent with alternatives to being home alone after school, such as participation in organised activities. | | | |
|  | |  |  | | | |
|  | | | ***Relationship Quality*** | | |  |
|  | | 1 | Parents should be aware that a close, supportive relationship with their adolescent determines how effective their efforts are in protecting their adolescent from alcohol-related harms. | | | |
|  | | 3 | Parents should be aware that a close, supportive relationship with their adolescent increases the likelihood that their adolescent will delay drinking. | | | |
|  | |  |  | | | |
|  | |  | ***Family Conflict*** | | | |
|  | | 2 | Parents should be aware that conflict in the home increases the risk that their adolescent will misuse alcohol. | | | |
|  | | 2 | Parents should avoid actions and statements that their adolescent could interpret as rejection. | | | |
|  | | 2 | Parents should not cause their adolescent to feel ashamed. | | | |
|  | |  |  | | | |
|  | |  | ***Parental Support*** | | | |
|  | | 3 | Parents should be aware that adolescents experiencing high stress are at an increased risk of alcohol misuse. | | | |
|  | |  |  | | | |
|  | |  | ***Parental Involvement*** | | | |
|  | | 1 | Parents should not criticise their adolescent harshly or cause them to feel ashamed. | | | |
|  | | 1 | Parents can be involved with their adolescent by trying to spend at least 15 minutes a day of one-on-one time with the adolescent. | | | |
|  | | 1 | Parents can be involved with their adolescent by helping their adolescent with their homework. | | | |
|  | | 1 | Parents can be involved with their adolescent by spending at least 15 minutes a day talking with the adolescent about things that are important to the adolescent. | | | |
|  | | 1 | Parents should ask their adolescent to tell their friends that they expect their support in situations involving alcohol, and to not pressure them to do anything they don’t want to do. | | | |
|  | |  |  | | | |
|  | |  | ***General communication*** | | | |
|  | | 2 | Parents should encourage communication with their adolescent by making themselves available to listen to their adolescent whenever they need it. | | | |
|  | | 3 | Parents should not criticise their adolescent harshly. | | | |
|  | | 3 | Parents should encourage communication with their adolescent by talking to children separately about important topics, if they have more than one child. | | | |
|  | |  |  | | | |
|  | |  |  | | | |
|  | |  | ***Peer Influence*** | | | |
|  | | 1 | Parents should get to know their adolescent’s friends and learn about their attitudes about drinking. | | | |
|  | | 1 | Parents should know their adolescent’s friends’ phone numbers and addresses. | | | |
|  | | 1 | Parents should get to know the parents of their adolescent’s friends. | | | |
|  | | 1 | Parents should communicate with the parents of their adolescent’s friends about the adolescents' activities. | | | |
|  | | 1 | Parents should find out other parents' family rules. | | | |
|  | | 1 | Parents should find out what other parents’ rules are relating to alcohol. | | | |
|  | | 1 | Parents should encourage their adolescent to spend time with friends who don’t drink. | | | |
|  | | 1 | Parents should encourage their adolescent to develop friendships with peers who don't drink. | | | |
|  | | 1 | Parents should encourage their adolescent to develop friendships with peers who do not drink, and who are otherwise a healthy influence. | | | |
|  | | 1 | Parents should encourage their adolescent to develop friendships with children who have family rules relating to alcohol. | | | |
|  | | 1 | Parents should find ways to encourage their adolescent to spend time with friends that the parent approves of, for example by inviting these friends to family gatherings. | | | |
|  | | 1 | If they are concerned about the negative influence of their child’s friends, parents should encourage their adolescent to take part in more structured, supervised activities. | | | |
|  | | 2 | Parents should encourage their adolescent to obtain the support of like-minded friends when faced with peer pressure to drink alcohol. | | | |
|  | | 2 | Parents should tell their adolescent that standing up for themselves in situations involving alcohol can earn them the respect of their peers. | | | |
|  | | 2 | Parents should encourage their adolescent to avoid developing friendships with peers who experiment with alcohol and other drugs. | | | |
|  | | 3 | Parents should encourage the parents of their adolescent’s friends to contact them with any concerns they may have about their adolescent’s behaviour. | | | |
|  | |  |  | | | |
|  | |  | ***Preparation for situations involving alcohol*** | | | |
|  | | 1 | When talking to their adolescent about situations involving alcohol, parents should role-play with the adolescent different strategies for handling these situations. | | | |
|  | | 1 | Parents should let their adolescent know that whilst refusing alcohol may be difficult at first, it will become easier the more they do it. | | | |
|  | | 1 | Parents should practice refusal techniques with their adolescent until they are comfortable saying "no". | | | |
|  | | 1 | Parents should tell their adolescent that when being pressured to drink, they can accept it but not drink it. | | | |
|  | | 2 | When talking to their adolescent about situations involving alcohol, parents should not tell the adolescent what to do in these situations, but help them to see choices and consequences for their actions. | | | |
|  | | 3 | Parents should let their adolescent know that whilst refusing alcohol may be difficult at first, it will become easier the more they do it. | | | |
|  | |  |  | | | |
|  | |  | ***Activities & Community Action*** | | | |
|  | | 1 | Parents should become involved in community activities aimed at the prevention of adolescent alcohol misuse. | | | |
|  | | 1 | Parents should encourage the parents of their adolescent’s friends to become involved in community activities aimed at the prevention of adolescent alcohol misuse. | | | |
|  | | 3 | Parents should be aware that adolescents who participate in activities that complement their interests and abilities are less likely to misuse alcohol. | | | |
|  | | 3 | Parents should report any alcohol outlets that they suspect serve alcohol to people who are underage. | | | |
|  | | |  | | |  |
|  | | | ***Parties*** | | |  |
|  | | 1 | Parents should not allow their adolescent to attend parties where there will be alcohol whilst they are under the age of 15. | | | |
|  | | 1 | Parents should not allow their adolescent to attend parties where there will be alcohol whilst they are under the age of 18. | | | |
|  | | 1 | When their adolescent is attending a party, parents should provide the adult supervising the party with their contact details in case a problem occurs. | | | |
|  | | 1 | Parents should volunteer to help at organised social events that their adolescent attends. | | | |
|  | | 1 | If their adolescent is over 15 and wants to drink at parties, parents should tell the adolescent that they expect them to wait until they are 18 before they start drinking. | | | |
|  | | 1 | If their adolescent is over 15 and wants to drink at parties, parents should not give the adolescent alcohol to take to parties. | | | |
|  | | 1 | If their adolescent is over 15 and wants to drink at parties, parents should provide the adolescent with alcohol when they go to parties and gatherings, so that they can monitor how much the adolescent drinks. | | | |
|  | | 1 | If their adolescent is over 15 and wants to drink at parties, parents should seek advice from other parents if unsure as to whether or not to supply alcohol to the adolescent for a party or gathering. | | | |
|  | | 1 | If their adolescent is over 15 and wants to drink at parties, parents should set rules for the adolescent about how much alcohol the adolescent is allowed to drink whilst at the party. | | | |
|  | | 1 | If their adolescent is over 15 and wants to drink at parties, parents should make an agreement with the adolescent about how much alcohol the adolescent is allowed to drink whilst at the party. | | | |
|  | | 1 | When hosting an adolescent party, parents should hire a security firm if there will be a large number of guests. | | | |
|  | | 1 | When hosting an adolescent party, parents should register the party with the police. | | | |
|  | | 1 | Parents should not allow alcohol consumption at any parties for adolescents under the age of 18. | | | |
|  | | 1 | If the parents decide to allow alcohol, they should discuss the rules regarding alcohol with their adolescent before inviting people to the party. | | | |
|  | | 1 | When hosting an adolescent party where alcohol consumption will be allowed parents should ensure that parents of guests are informed that there will be alcohol at the party, as well as what restrictions will be in place to prevent guests from misusing alcohol. | | | |
|  | | 1 | When hosting an adolescent party where alcohol consumption will be allowed parents should make it clear to their adolescent and their guests that drunkenness will not be tolerated. | | | |
|  | | 1 | When hosting an adolescent party where alcohol consumption will be allowed parents should confiscate alcohol if necessary. | | | |
|  | | 1 | When hosting an adolescent party where alcohol consumption will be allowed parents should ensure that they have a strategy in place for anyone who drinks too much. | | | |
|  | | 1 | When hosting an adolescent party where alcohol consumption will be allowed parents should know first aid responses for alcohol intoxication. | | | |
|  | | 1 | When hosting an adolescent party where alcohol consumption will be allowed parents should ensure that a responsible adult who knows first aid is present at the party. | | | |
|  | | 1 | When hosting an adolescent party where alcohol consumption will be allowed parents should not allow guests who have been drinking to drive home. | | | |
|  | | 1 | When hosting an adolescent party where alcohol consumption will be allowed parents should make sure that guests have a safe ride to and from the party with a responsible adult. | | | |
|  | | 1 | When hosting an adolescent party where alcohol consumption will be allowed, parents should put in place strategies to prevent guests from misusing alcohol. | | | |
|  | | 1 | In order to limit the amount of alcohol consumed by guests, parents should not allow guests to bring their own alcohol. | | | |
|  | | 1 | In order to limit the amount of alcohol consumed by guests, parents should ask guests to hand over any alcohol that they bring, and confiscate any alcohol that is not handed over. | | | |
|  | | 1 | In order to limit the amount of alcohol consumed by guests, parents should only serve alcohol upon request. | | | |
|  | | 1 | In order to limit the amount of alcohol consumed by guests, parents should allocate one responsible adult as the designated server of alcohol. | | | |
|  | | 1 | In order to limit the amount of alcohol consumed by guests, parents should serve the alcohol from one location, to help control the amount of alcohol consumed. | | | |
|  | | 1 | In order to limit the amount of alcohol consumed by guests, parents should provide one glass of champagne or wine to celebrate, and then serve only soft drinks for the rest of the party. | | | |
|  | | 1 | In order to limit the amount of alcohol consumed by guests, parents should provide a predetermined, maximum amount of alcohol per guest. For example, one or two standard drinks. | | | |
|  | | 1 | In order to limit the amount of alcohol consumed by guests, parents should enforce a rule of no more than one drink an hour rule for guests. | | | |
|  | | 1 | In order to limit the amount of alcohol consumed by guests, parents should restrict the time during which alcohol will be served. | | | |
|  | | 1 | In order to limit the amount of alcohol consumed by guests, parents should only serve alcoholic drinks in small glasses. | | | |
|  | | 1 | In order to limit the amount of alcohol consumed by guests, parents should not ‘top up’ drinks. | | | |
|  | | 1 | In order to limit the amount of alcohol consumed by guests, parents should only serve low-alcohol alcoholic drinks. | | | |
|  | | 1 | In order to limit the amount of alcohol consumed by guests, parents should premix any spirits to be served. | | | |
|  | | 1 | In order to limit the amount of alcohol consumed by guests, parents should discourage guests from drinking from stubbies or cans. | | | |
|  | | 1 | In order to limit the amount of alcohol consumed by guests, parents should provide plenty of food. | | | |
|  | | 1 | In order to limit the amount of alcohol consumed by guests, parents should ensure there are plenty of non-alcoholic drinks available, such as soft drinks and fruit juice. | | | |
|  | | 1 | In order to limit the amount of alcohol consumed by guests, parents should ensure there are more non-alcoholic drinks available than alcoholic drinks. | | | |
|  | | 1 | In order to limit the amount of alcohol consumed by guests, parents should provide a range of attractive non-alcoholic drinks such as alcohol-free cocktails. | | | |
|  | | 1 | In order to limit the amount of alcohol consumed by guests, parents should only serve soft drink in cans and bottles. | | | |
|  | | 1 | In order to limit the amount of alcohol consumed by guests, parents should avoid serving drinks that can be tampered with, such as punch and soft drinks in cups. | | | |
|  | | 1 | In order to limit the amount of alcohol consumed by guests, parents should enforce a rule that guests cannot bring soft drinks that have already been opened, as these may have spirits already added to them. | | | |
|  | | 1 | In order to limit the amount of alcohol consumed by guests, parents should ensure that drinking is not the main focus of the party. | | | |
|  | | 2 | Parents should not allow their adolescent to attend any parties that are not adequately supervised. | | | |
|  | | 2 | Parents should not allow their adolescent to attend an adolescent party where there will be alcohol, whilst they are under the age of 18. | | | |
|  | | 2 | Parents should not allow alcohol consumption at any parties for adolescents over the age of 15, but under the age of 18. | | | |
|  | | 2 | When hosting an adolescent party, parents should discuss with other parents whether or not to allow alcohol at the party. | | | |
|  | | 2 | When hosting a party for adolescents over the age of 15, parents should discuss with other parents whether or not to allow alcohol at the party. | | | |
|  | | 2 | When hosting an adolescent party, parents should not drink any alcohol themselves. | | | |
|  | | 3 | Parents should not allow their adolescent to attend an adolescent party where there will be alcohol, whilst they are under the age of 15. | | | |
|  | |  |  | | | |
|  | | |  | | |  |
|  | | | ***When an adolescent has been drinking without permission*** | | |  |
|  | | 1 | If parents are concerned that their adolescent may be misusing alcohol they should confirm their suspicions by gathering evidence before talking to the adolescent directly about their drinking. | | | |
|  | | 1 | If their adolescent comes home affected by alcohol when they are not allowed to drink, parents should address the situation as soon as possible. | | | |
|  | | 1 | If their adolescent comes home after drinking without permission parents should try to find out how much alcohol has been consumed. | | | |
|  | | 1 | If parents are concerned that their adolescent may be misusing alcohol they should ask their adolescent where they got the alcohol from. | | | |
|  | | 1 | Following an incident where their adolescent child has misused alcohol, parents should talk to the adolescent about how they can earn back their parent’s trust. | | | |
|  | | 1 | Following an incident where their adolescent child has misused alcohol, parents should monitor the adolescent more closely. | | | |
|  | | 1 | Following an incident where their adolescent child has misused alcohol, parents should not monitor the adolescent more closely. | | | |
|  | | 1 | Following an incident where their adolescent child has misused alcohol, parents should try not to blame themselves for the adolescent’s drinking behaviour. | | | |
|  | | 1 | If the adolescent is drinking alcohol for the excitement, parents should suggest alternatives to alcohol, such as rock climbing or mountain biking, in order to discourage their child from misusing alcohol again. | | | |
|  | | 2 | Parents should be aware that adolescents may deliberately dress to look older to get into licensed venues. | | | |
|  | | 2 | If their adolescent comes home drunk, parents should check on them throughout the night to ensure they don’t roll onto their back. | | | |
|  | | 2 | When approaching their adolescent about their alcohol misuse, parents should tell the adolescent the risks associated with alcohol use. | | | |
|  | | 3 | If their adolescent comes home drunk, parents should help them to bed and lay them on their side to prevent them from choking if they vomit. | | | |
|  | | 3 | If their adolescent comes home *very* drunk, parents should help them to bed and lay them on their side to prevent them from choking if they vomit. | | | |
|  | | 3 | If their adolescent comes home *very* drunk, parents should check on them throughout the night to ensure they don’t roll onto their back. | | | |
|  | | 3 | Following an incident where their adolescent child has misused alcohol, parents should get professional help if they think the adolescent may have a problem with alcohol. | | | |
