# Supplementary material for: Parenting strategies for reducing adolescent alcohol use: a Delphi consensus study
Source: BMC Public Health. 2011 Jan 6;11:13. doi: 10.1186/1471-2458-11-13 (PMC3022696; doi:10.1186/1471-2458-11-13)
Supplement: Additional file 2 — Parenting guideline for adolescent alcohol use. Final document of parenting strategies for preventing or reducing adolescent alcohol use. [file 1471-2458-11-13-S2.PDF]

Parenting Guidelines
for Adolescent Alcohol Use

Purpose of these guidelines

These guidelines are designed to help parents prevent or reduce their adolescent child's alcohol use, as recommended by the NHMRC *Australian Guidelines to Reduce Health Risks from Drinking Alcohol* (2009).

How these guidelines were developed

The following guidelines are based on a systematic review of high quality research evidence and/or the opinions of a panel of 32 Australian experts with a minimum of five years experience in one of the following: treatment of adolescents with alcohol problems, research regarding adolescent drinking and parenting practices, drug and alcohol education for adolescents and/or parents. Details of the methodology can be found in: Ryan et al. (In press). Parenting to reduce adolescent alcohol consumption: A Delphi consensus study. *BMC Public Health*. The guidelines were the product of a collaboration between Orygen Youth Health Research Centre (University of Melbourne), Turning Point Alcohol and Drug Centre (Monash University and Eastern Health), and the Australian Drug Foundation, with funding from VicHealth.

How to use these guidelines

These guidelines are a general set of recommendations regarding how you as a parent can delay or reduce your child's alcohol consumption. Each family is unique and it is important that you adapt the information provided in these guidelines to your situation. These guidelines were developed for preventing or reducing adolescent alcohol consumption in Australian families. While many of these strategies are likely to be relevant in other communities, they may need to be adapted for other cultural groups or countries.

To help you identify guidelines that are relevant to your family, we recommend that you complete the Survey at **www.parentingstrategies.net** *before* reading these Guidelines.

Although these guidelines are copyright, they can be freely reproduced for non-profit purposes provided the source is acknowledged.
Please cite these guidelines as follows: Parenting Strategies Program (2010). *Parenting Guidelines for Adolescent Alcohol Use*. Melbourne: Orygen Youth Health Research Centre, University of Melbourne.

Enquiries should be sent to:

enquiries@parentingstrategies.net

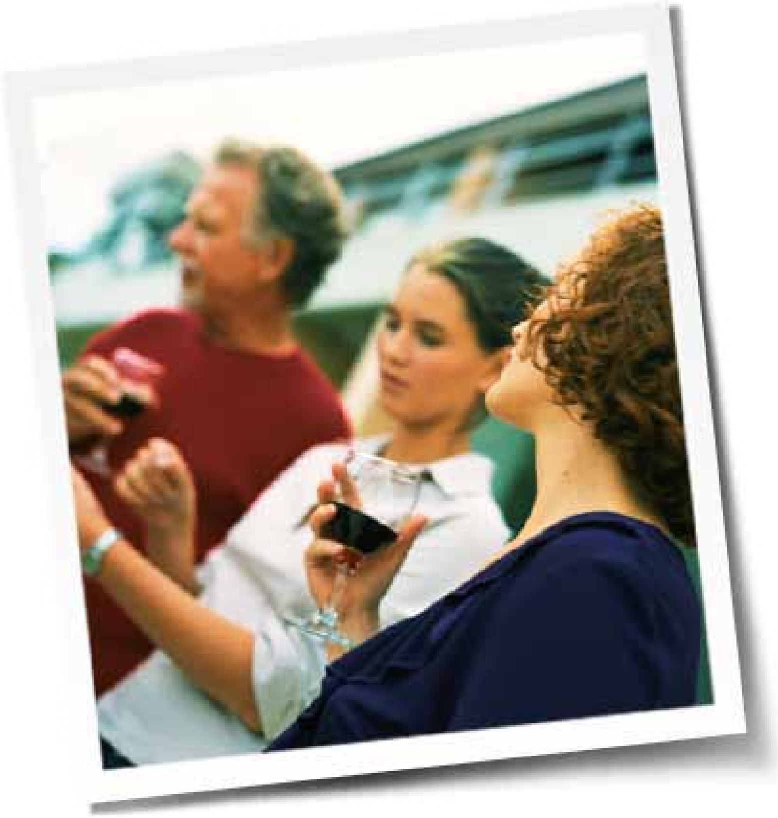

You can influence your adolescent's choices about alcohol

As a parent you have a major influence on your adolescent child's drinking behaviour and you can help prevent them from drinking alcohol or from harmful use. Your influence on your adolescent's attitudes and decisions about alcohol is greatest before they start drinking. This guide describes a range of strategies you can use to prevent your adolescent from misusing alcohol. When choosing from these strategies, select approaches that are appropriate for your adolescent's maturity and personality.

Some things you should know about adolescent drinking

Risks associated with adolescent drinking

Alcohol is a drug. It acts as a depressant (slows down the central nervous system) and has numerous other effects on the body. Adolescents have less physical tolerance to the effects of alcohol. Adolescence is a time when the brain is still rapidly developing and it is therefore more susceptible to damage due to drinking alcohol. There are a number of other harms associated with alcohol that are more likely to occur during adolescence (see box on *Alcohol-related risks in adolescence*).

Over 80% of adolescents have used alcohol by the time they are 14 years of age.[1] Research shows that the earlier an adolescent starts drinking, the greater the chance that they will have problems with alcohol later in life. Because of the health risks associated with drinking, the National Health and Medical Research Council has recommended that adolescents under the age of 15 do not drink any alcohol at all, and that adolescents between the ages of 15 and 17 delay starting to drink for as long as possible. Any drinking by adolescents under the age of 18 should be at low risk levels, in a safe environment, and supervised by an adult (see box on *Australian Guidelines to Reduce Health Risks from Drinking Alcohol*).

What does the law say about adolescent alcohol consumption?

There are laws in every state and territory restricting the provision of alcohol to people under the age of 18. You need to be aware of the current laws in your state. You can find information about the laws applying to your state or territory at **www.reachout.com.au**

Why do adolescents drink alcohol?

Although it may sometimes seem like it, adolescents do not simply drink alcohol to disobey their parents. Experimentation and risk taking are a normal part of adolescent behaviour, which includes drinking. When adolescents drink, they often hide it from their parents. As your adolescent child gets older, the likelihood they will drink alcohol increases.

There are many factors that may influence an adolescent's decision to drink:

- Many adolescents associate alcohol use with becoming an adult
- Drinking may be considered normal within their peer or cultural groups
- Portrayal and marketing of alcohol in the media may encourage drinking
- Parents' use of and attitudes about alcohol also influence drinking

Alcohol-related risks in adolescence

Short-term problems caused by alcohol intoxication

Alcohol intoxication in young people can result in significant harms, including:

Physical injuries and risk-taking. Adolescents who binge drink (drink a large amount in a short space of time) are more likely to engage in risky behaviours that can result in serious injury, such as falls and road traffic accidents. Adolescents who drink are also more likely to engage in risky sexual activity, exposing themselves to sexually transmitted infections (such as chlamydia). Alcohol contributes to the three leading causes of death among young people (unintentional injuries, homicide and suicide).

Violence and sexual assaults. Young people who drink heavily are more likely to become involved in violent confrontations, either as aggressors or victims. Both boys and girls are more vulnerable to sexual assault when intoxicated with alcohol.

Suicide and self-injury. Risk of suicide and self-injury is higher in adolescents who drink heavily or frequently. Alcohol increases the risk in several ways. It can intensify feelings of anxiety, depression and aggression, while at the same time undermining the person's ability to cope. It can also make a person more likely to act on suicidal thoughts.

Long-term problems as a result of adolescent alcohol use

Adolescents who drink are at a greater risk of a broad range of psychological and physical problems in adulthood. These include:

Alcohol abuse/dependence. Evidence indicates that any drinking during adolescence increases the chance of developing a drinking problem in early adulthood, and the earlier someone starts drinking the greater the risk.

Depression and other mental illnesses. Young people who suffer from mental illnesses are more likely to use alcohol to help them cope. While alcohol can make people feel better in the short term, heavy drinking can make these problems worse.

Social problems. Abuse of alcohol is associated with dropping out of school, unemployment and social isolation.

Physical health problems. Persistent heavy drinking can produce a range of physical health problems, including liver disease and brain damage.

Sources:
National Health & Medical Research Council (2009). *Australian Guidelines to Reduce Health Risks from Drinking Alcohol.* Canberra.
Ellickson, P. L., Tucker, J. S., & Klein, D. J. (2003). Ten-Year Prospective Study of Public Health Problems Associated With Early Drinking. *Pediatrics*, *111*(5), 949-955.
Moore, E., Coffey, C., Carlin, J. B., Alati, R., & Patton, G. C. (2009). Assessing alcohol guidelines in teenagers: results from a 10-year prospective study. *Australian and New Zealand Journal of Public Health, 33*(2), 154-159.

[1]White, V., & Hayman, J. (2006). *Australian secondary school students' use of alcohol in 2005.* Melbourne: Centre for Community Child Health, Royal Children's Hospital.

There are also a number of factors that increase the risk of an adolescent drinking heavily:

- Experiencing emotional or psychological problems
- Not feeling connected to family, school or community
- Behaviour problems
- Family history of alcohol problems

When adolescents drink, they often drink with the aim to get drunk, and are more likely to binge drink. Although binge drinking is common among Australian adolescents, any episode of binge drinking is still a cause for concern. However, a single episode of binge drinking does not necessarily mean your adolescent has an alcohol problem.

Delay your adolescent's introduction to drinking alcohol

Early introduction to drinking (before the age of 15) is associated with a number of alcohol-related problems later in life (see box on *Alcohol-related risks in adolescence*).

The longer your adolescent delays alcohol use, the less likely they are to develop problems associated with alcohol. You should therefore aim to keep your adolescent child from experimenting with alcohol for as long as possible. To do this, do not give them any alcohol while they are under the age of 15, and delay their first alcoholic drink for as long as possible. You can teach your adolescent about responsible drinking without necessarily allowing them to drink. If they express an interest in trying alcohol when they are over the age of 15, explain to them why it is best to wait until they are older to start drinking.

Model responsible drinking and attitudes towards alcohol

Parents are important role models for their children, even during adolescence. Your attitude towards alcohol, what you drink, how much, when and where you drink are all a major influence on whether or how your adolescent will drink in the future.

Australian Guidelines to Reduce Health Risks from Drinking Alcohol

Guideline 1: Reducing the risk of alcohol-related harm over a lifetime
For healthy men and women, drinking no more than two standard drinks on any day reduces the lifetime risk of harm from alcohol-related disease or injury.

Guideline 2: Reducing the risk of injury on a single occasion of drinking
For healthy men and women, drinking no more than four standard drinks on a single occasion reduces the risk of alcohol-related injury arising from that occasion.

Guideline 3: Children and young people under 18 years of age
A: Parents and carers should be advised that children under 15 years of age are at the greatest risk of harm from drinking and that for this age group, not drinking alcohol is especially important.
B: For young people aged 15-17 years, the safest option is to delay the initiation of drinking for as long as possible.

Guideline 4: Pregnancy and breastfeeding
A: For women who are pregnant, not drinking is the safest option.
B: For women who are breastfeeding, not drinking is the safest option.

What is a standard drink?
A standard drink contains 10 grams of alcohol, for example 100ml of wine, 30ml of spirits or 285ml of heavy beer.

Adapted from: National Health & Medical Research Council (2009). *Australian Guidelines to Reduce Health Risks from Drinking Alcohol*. Canberra.

This influence begins at a very early age.Warning your adolescent about the dangers of drinking will not be effective if you do not set a good example yourself. If you drink, you should model responsible drinking by establishing and following your own rules for drinking responsibly (see box on *Tips for modelling responsible drinking*) Explain these rules to your adolescent.

Talk to your child about alcohol

Talking to your child about the risks associated with alcohol can help reduce their risk of alcohol-related harms. Even if your adolescent chooses not to drink, you should still discuss alcohol with them.

Preparing for the talk

Before talking to your child, take some time to prepare for the conversation. Make sure you are knowledgeable about alcohol and its effects and try to find out how alcohol is addressed in your child's school curriculum. Evaluate your own behaviours and attitudes regarding alcohol, and clarify your attitudes and beliefs about adolescent drinking. For example, is there a certain age when you think it is acceptable for an adolescent to try alcohol? Think about the main points you want to discuss with your child, questions they may ask, and how you will respond to them. You should be prepared to answer some difficult questions.

How to talk to your child about alcohol

When talking to your adolescent about alcohol, think about previous occasions when you have related well with them and try to use these methods. Choose a time when both of you are relaxed. Make it a conversation, not a lecture. Think about what you convey through your tone of voice, facial expression and body language. Remain calm and try not to

express judgment or respond with anger if you hear something you don't like. Try not to bring up the adolescent's past mistakes during the conversation.

Tailor information and language to your adolescent's age and maturity and ask questions to make sure they understand what you are saying. Listen without interrupting when your child speaks and show them you are listening by nodding, asking questions, or repeating phrases back to them. If you can't answer a question, admit it and look for the answer from a reputable source such as a book, reliable website (see on box *Reputable websites to find information about alcohol*), or health professional.

What to talk about

You should not present a permissive approach to alcohol when talking to your adolescent, as this can increase the likelihood that they will misuse alcohol. Tell your child the facts about alcohol, its harms, and the health benefits of choosing not to drink. Explain to them that their brain is still developing and is therefore more vulnerable to harm caused by alcohol. Talk about how the effects of alcohol vary between individuals, depending on the amount of alcohol, the person and the situation. Teach them that different types of alcoholic drinks contain different amounts of alcohol and ensure they know the laws relating to underage alcohol consumption, drunkenness and drink driving.

Emphasise the short-term harms associated with alcohol, as these are generally of greater concern to adolescents (see box on *Alcohol-related risks in adolescence*). For example, explain to them that alcohol may cause them to do something embarrassing that might damage their self-esteem and friendships. Talk about the positive as well as the negative effects of alcohol, and avoid scare tactics or exaggerating its negative effects.

Discuss with your child their perceptions of alcohol

Ask your child what they think about alcohol and encourage them to talk about anything that interests or concerns them about drinking. Ask them why they think young people drink and listen carefully to their response. Address any myths or misinformation your child may have about alcohol. Encourage them to question the assumption that most adolescents drink, help them realise that many of their peers are not drinking, and address the myth that most adolescents get drunk. Talk about how alcohol is portrayed in the media, highlighting how alcohol is glamourised and how the media spreads myths about drinking.

Tips for modelling responsible drinking:

- Limit your alcohol use, especially in front of your children
- Do not get drunk, especially in front of your children
- Sometimes decline the offer of alcohol
- Provide food and non-alcoholic beverages if making alcohol available to guests
- Never drink and drive
- Do not let other adults drive after they have been drinking
- Do not convey to your children the idea that alcohol is fun or glamorous through stories about your own or others' drinking
- Do not portray alcohol as a good way to deal with stress, such as by saying, "I've had a bad day, I need a drink!"
- Use healthy ways to cope with stress without alcohol, such as exercise, listening to music, or talking things over

Discuss your expectations about their alcohol consumption

With children and younger adolescents

Discuss with your child what your expectations are regarding their alcohol consumption. Be clear, direct and specific. Give them valid reasons why they should not drink. Talk about how the best way for them to avoid the harms associated with alcohol is to not drink at all before the age of 15, and even better, to wait until they are 18.

With older adolescents

Discuss how, if your adolescent does drink, they should do so in moderation. Explain your expectations for specific situations, such as at family celebrations, adolescent parties or "Schoolies Week".

When talking to your adolescent child about alcohol, discuss with them how risks associated with alcohol can be minimised. Tell them not to participate in potentially risky activities, such as skateboarding, swimming or riding a bike after they have consumed alcohol. Also discuss the added risks of using alcohol with other drugs.

Continue to talk to your child about alcohol throughout their adolescence. Discuss with them how, if they choose to drink when they are an adult, they should do so responsibly.

Establish family rules

Developing general family rules

Having clear family rules, including those not specific to alcohol, is important in protecting your adolescent from alcohol misuse. When formulating rules and consequences for your adolescent, try to support their growing independence, while setting appropriate limits, and use positive reinforcement where possible. Involve your adolescent in developing family rules for them to follow. Once established, make sure the family is clear on exactly what the rules are and that each member understands them. Be prepared to negotiate on rules regarding minor matters, but do not change the family rules or consequences without first discussing it with your adolescent. Review rules as your adolescent shows more maturity and responsibility.

Parents should support each other regarding family rules and present a united front in enforcing them. Make sure your adolescent understands that family rules are to be maintained when they are away from the family home and that you expect them to make wise choices based on family rules.

Rules about alcohol

Rules about alcohol are important in protecting your adolescent from alcohol misuse. Establish these rules for your adolescent before they are exposed to situations involving alcohol. Establish rules regarding alcohol for when your adolescent is at home unsupervised. Tell them that any alcohol stored in the family home is strictly off-limits to them and their friends. Make sure they know that the rules about alcohol are a protective measure and not just a restriction on their freedom.

Consequences for when rules are broken

Establish realistic consequences for when family rules are broken. These consequences should be harsh enough to be a deterrent, but not so harsh that they may damage your relationship with your adolescent should they be imposed. Try to involve your adolescent in the development of consequences and make sure they are very clear about what these consequences are.

Enforce established consequences consistently every time that family rules are broken. When enforcing established consequences, calmly explain to your adolescent why their behaviour has resulted in that consequence and make sure that they know that they are loved.

Seek help with parenting if your adolescent continues to break rules regarding alcohol.

Reputable websites to find information about alcohol

Australian Drug Foundation
http://www.adf.org.au

The Australian Drug Foundation (ADF) is an independent, non-profit organisation working to prevent and reduce alcohol and drug problems in the Australian community. Its website is a good source of factual information on most types of drugs used illegally or unsafely. There is an online shop with pamphlets, books and videos.

Australian Drug Information Network (ADIN)
http://www.adin.com.au

This site is funded by the Australian Department of Health and Ageing to provide a central point of access to Australian drug and alcohol information.

National Drug and Alcohol Research Centre (NDARC)
http://www.med.unsw.edu.au/ndarc

NDARC is based at the University of New South Wales and is funded by the Commonwealth Government. Its aim is to increase the effectiveness of treatment for drug and alcohol problems in Australia. Its website has information and online ordering for some excellent booklets on alcohol and other drugs.

Between the Lines
http://www.betweenthelines.net.au

This website is aimed at young people who use or are curious about using alcohol or other drugs, or are concerned about a friend's use. It includes information about different drugs, where to seek help (including what help is available) and the risks associated with drug use.

Somazone
http://www.somazone.com.au

This website is aimed at young people and has information about alcohol and other drugs, mental illness, relationships and family issues. Young people post stories about their own lives and can get feedback from other visitors to the site, and can ask questions which are answered by health professionals and other volunteers with good knowledge of the relevant issues.

Turning Point Alcohol & Drug Centre
http://www.turningpoint.org.au

Turning Point is a centre that provides treatment and research focussed on alcohol and other drugs. The website provides access to an online counselling service and details of 24 hour helplines for young people and families for help with problems associated with alcohol and other drugs.

Monitor your adolescent when you are not around

Adolescents are more likely to misuse alcohol when adults are not around. Monitoring your adolescent's whereabouts and activities when they are unsupervised reduces the likelihood that they will misuse alcohol. Monitoring refers to knowing about your adolescent's activities, whereabouts and friends. You should especially know where they are if they or others may be drinking.

Before your adolescent goes out, you should:

- Ask them where they will be, what they will be doing, and who they will be with
- Set a curfew and know what time to expect them home
- Make arrangements with them about how they will get home safely
- Ask them to contact you if their plans change
- Make sure they have a way to contact you

If giving them money, discuss how much they will need and how it will be spent.

Tell your adolescent that you are monitoring their activities not because you are nosey, but because you care about their safety. Most adolescents appreciate their parents monitoring their activities and see it as proof of their parents' concern for their wellbeing.

Be aware that although monitoring your adolescent's activities is important in protecting them from alcohol misuse, being overly strict or harsh may cause

your adolescent to rebel by misusing alcohol. Try to balance monitoring with your adolescent's need for privacy and adjust your monitoring as your adolescent matures to encourage their growing independence.

Prepare your adolescent to deal with the influence of peers

Peers are a major influence during adolescence
As your child approaches adolescence, friends and "fitting in" becomes very important, but obtaining acceptance from peers can be difficult. Your adolescent's friends are a major influence on their decisions about alcohol. Your adolescent is more likely to drink if their friends do. However, despite the growing influence of peers, you can still have a positive influence on your adolescent's alcohol use. A good relationship with your adolescent will reduce any negative influence from their friends.

Encourage positive friendships
Get to know your adolescent's friends. Encourage your adolescent to invite their friends over when you are at home. This will allow you to get to know your adolescent's friends better and help you learn about your adolescent's activities. Talk to your adolescent's friends and try to interact with them. Talk to your adolescent about qualities that really count in a friend, such as being kind and trustworthy, rather than popular and "cool".

Enlist the support of other parents
Build a support network with other parents. The families of your adolescent's friends may have different values and attitudes regarding alcohol to yours and this may cause some difficulty in maintaining rules regarding alcohol for your adolescent.

Dealing with peer pressure to drink
Your adolescent may find themselves in situations where it is difficult for them to say no to alcohol, because of peer pressure. Try to prepare them by focussing on specific situations that they may encounter and talk about different ways they can deal with peer pressure to drink. Tell your adolescent that the decision whether or not to drink is theirs, and not their friends'. Help them develop ways to say no to offers of alcohol before they are faced with situations where this may occur. Remember that peers can also be a positive influence. Encourage your adolescent to support others who experience peer pressure to use alcohol.

Unsupervised adolescent drinking

Know the range of settings in which unsupervised adolescent drinking occurs, as well as the range of sources that adolescents can obtain alcohol from. Be aware that the risks are greater when adolescents drink unsupervised in public places such as playgrounds or car parks.

Prepare your adolescent for situations where others misuse alcohol
Discuss with your adolescent situations they may be faced with where other people are misusing alcohol. Help your adolescent to develop strategies for handling or removing themselves from situations involving alcohol misuse. Make it clear to your adolescent the ways in which you will support them, such as offering to pick them up. Tell your adolescent to call you if ever faced with a situation involving alcohol and assure them that, whatever the circumstances, you will pick them up. Talk about ways to minimise any potential embarrassment that may be associated with getting picked up.

Discuss drink spiking and other dangers
Discuss strategies for minimising harm associated with alcohol when your adolescent is out with friends, such as sticking with their friends, not walking off alone, and ensuring that others know where they are. Talk to them about the dangers of drink spiking, for example, how someone can deliberately put a dangerous amount of alcohol in a drink with the aim of intoxicating another person, and how even non-alcoholic drinks can be spiked with alcohol. Discuss how they can protect themselves against drink spiking.

Warn about drink driving
Warn your adolescent about the dangers of getting into a car driven by someone who has been drinking. Make it a rule that your adolescent must never get into a car driven by someone who has been drinking. When your adolescent is going out, talk to the person driving and confirm that they will not be drinking. Establish a plan for when your adolescent is faced with a drink driver, such as agreeing to pay for a taxi or picking them up. Let your adolescent know that there will be no negative repercussions for calling you for a lift when faced with a situation involving a drink driver. Establish and enforce a rule that your adolescent must never drink alcohol and drive.

Protect your adolescent from alcohol misuse at parties
Providing your adolescent with alcohol for parties and gatherings increases the likelihood that they will misuse alcohol. Never supply alcohol to your adolescent's friends. Do not allow your adolescent to attend a party that is not adequately supervised. Before allowing them to attend, contact the party host to confirm whether it will be adequately supervised. For example, find out:

- Will a responsible adult be there?
- Will alcohol be allowed?
- Will alcohol consumption be regulated?
- Is the party invitation-only?
- What time will the party finish?

If you decide to let your adolescent attend, get the name and number of the responsible adult who will be supervising the party. Monitor your adolescent by checking in with the responsible adult. Make sure your adolescent has a safe ride with a responsible adult to and from the party.

Give positive feedback
It is important to give positive feedback when your adolescent follows the rules. If your adolescent has decided to call you to be picked up from a party or another situation where alcohol is being misused, make sure they know you are proud of them. Positive feedback increases the chances they will continue to follow the rules later.

When your adolescent has been drinking without permission

Despite your best efforts, you may not be able to prevent your adolescent from drinking. If they do, you should be concerned, but remember that many adolescents drink, yet few develop alcohol problems.
Know the signs that indicate your adolescent may be misusing alcohol, and do not ignore any concerns that you have (see box on *Warning signs that indicate*

your adolescent may be misusing alcohol). If your adolescent comes home drunk, wait until they are sober before talking to them about their behaviour. You should know how to respond if your adolescent has a medical emergency due to intoxication (see *Helping Someone with a Drinking Problem Mental Health First Aid Guidelines:* **www.mhfa.com.au/Guidelines.shtml**)

Talk to your adolescent about your concerns
When approaching your adolescent about their alcohol misuse, try to stay calm. Allow them to tell their side of the story and do not lecture them. Use dealing with your adolescent's alcohol misuse as an opportunity to maintain or improve communication between yourself and your adolescent. Find out as much as you can about your adolescent's understanding and beliefs about alcohol, and about how drinking makes them feel. Tell them what concerns you about their alcohol use, such as that they have broken the rules or that they have put their health and safety at risk. Take care to communicate that you disapprove of the behaviour, not the adolescent themselves. Use 'I' statements, such as 'I feel very upset about you drinking at that party' rather than you statements such as 'you are a lying, untrustworthy child'. If you are unsure of how to approach your adolescent about their alcohol misuse, consider enlisting the help of someone knowledgeable, such as a family doctor or a counsellor.

Warning signs that indicate your adolescent may be misusing alcohol

There are no warning signs that definitely indicate an adolescent is engaged in risky drinking. However, there are a range of signs and behaviours that, when seen in combination, may indicate an adolescent is drinking excessively. These signs include:

- Repeated health complaints
- Changes in sleeping patterns
- Changes in mood, especially irritability
- Starting arguments, withdrawing from the family or breaking family rules
- Dropping grades, frequent school absences or discipline problems at school
- Changes in social activities and social groups

These signs can also result from other physical and psychological problems. If you observe a number of these signs in your adolescent child, consider consulting your GP to rule out other potential causes.

Adapted from: American Academy of Child & Adolescent Psychiatry (2008). Teens: Alcohol and Other Drugs, *Facts for Families*. Washington, DC.

When hosting an adolescent party

Preparing to host an adolescent party
Establish and enforce a rule that your adolescent is to obtain your permission before holding a party or gathering in the family home or elsewhere. When hosting an adolescent party, consider advice provided by reputable "partysafe" websites such as those produced by state police or the Australian Drug Foundation (**www.adf.org.au**). Make sure the party is adequately supervised by one or more adults. You should know strategies for de-escalating potential violence (see box on *Strategies for de-escalating aggression*). You should also consider how you will prevent or manage gatecrashers. Plan age-appropriate activities together with your adolescent to take the focus off drinking at the party.

Decide whether to allow alcohol consumption
Never allow alcohol consumption at parties for adolescents under the age of 15. Before hosting a party for older adolescents, it is important to make a clear decision about whether or not to allow alcohol. When making this decision, be aware of the legal responsibilities of hosting an adolescent party where alcohol is consumed (in some parts of Australia it is illegal to provide alcohol to a person under 18; see **www.reachout.com.au**). If you have decided not to allow alcohol at the party, explain to your adolescent the reasons why.

How to prevent alcohol misuse at parties where alcohol is allowed
If you have decided to allow alcohol at a party for older adolescents, implement strategies to prevent alcohol misuse by guests (see reputable "partysafe" websites, as mentioned above). Discuss the rules regarding alcohol with your adolescent before inviting people to the party. Make sure that parents of guests are informed that there will be alcohol at the party, as well as the rules you will have in place to prevent alcohol misuse. Be prepared to confiscate alcohol if necessary, at least for the duration of the party (you may need to return the alcohol after the party in order to avoid an accusation of theft). Make it clear to your adolescent and guests that drunkenness will not be tolerated and have a strategy prepared for if someone drinks too much. Make sure a responsible adult who knows first aid is present at the party. You should also limit your own alcohol consumption.

Make sure that guests have a safe ride to and from the party with a responsible adult. Do not allow guests who have been drinking to drive home from the party.

Establish and maintain a good relationship with your adolescent child

A close and supportive relationship with your adolescent does not guarantee that they will not misuse alcohol, but it does reduce the likelihood. It also influences how effective your efforts are in protecting them from alcohol misuse and will increase the likelihood that they will seek help from you if they are faced with an issue regarding alcohol.

There are a number of things you can do to establish and maintain a good relationship with your adolescent, such as:

- Support them in pursuing their interests and in dealing with problems
- Show an interest and be involved in their life
- Work to create open communication between yourself and your adolescent
- Cultivate their trust by being consistent in following through on promises and enforcing rules
- Regularly demonstrate that you care about them
- Regularly tell them that you love them

Strategies for de-escalating aggression

If a person becomes aggressive, assess the risk of harm to yourself, the person and others. Ensure your own safety at all times so that you can continue to be an effective helper. If you feel unsafe, seek help from others. Do not stay if your safety is at risk. Remain as calm as possible and try to de-escalate the situation with the following techniques:

- Talk in a calm, non-confrontational manner
- Speak slowly and confidently with a gentle, caring tone of voice
- Try not to provoke the person; refrain from speaking in a hostile or threatening manner and avoid arguing with them
- Use positive words (such as “stay calm”) instead of negative words (such as “don’t fight”) which may cause the person to overreact
- Consider taking a break from the conversation to allow the person a chance to calm down
- If inside, try to keep the exits clear so that the person does not feel penned in and you and others can get away easily if needed

If violence has occurred, seek appropriate emergency assistance.

Support your adolescent in dealing with problems and stress

Support your adolescent in dealing with problems and coping with disappointment. Encourage them to discuss their problems and concerns with you. Give them a chance to solve their own problems, as this will help them build self-esteem, and encourage them to accept the consequences of their choices. Admit if you don’t have all the answers, as this will encourage their respect.

Encourage your adolescent to use healthy approaches for dealing with stress, like exercise, music or talking over problems. Monitor them for signs of high stress and mental health problems such as anxiety or depression, as adolescents with these problems are at an increased risk of alcohol misuse.

Help your adolescent feel good about themselves

There are a number of ways you can help your adolescent to feel good about themselves:

- Ensure that your positive comments outweigh your negative comments when you talk to them
- Praise them for good behaviour
- Praise them for their efforts as well as their achievements
- Do not tease them in a way that could be perceived as hurtful
- Avoid actions and statements that they are likely to interpret as rejection

Be involved in your adolescent’s life

Your adolescent is less likely to misuse alcohol if you are involved in their life in a positive way. Spending time with your adolescent facilitates communication between the two of you. Use the following strategies:

- Regularly spend one-on-one time giving them your undivided attention
- Try establishing a regular weekly routine for doing something special with your adolescent
- Find ways for your adolescent to be involved in family life, such as doing chores or caring for younger brothers or sisters
- Engage in activities together as a family on a regular basis
- Try to include your adolescent’s friends in family activities

Establish and maintain good communication with your adolescent

Good communication between you and your adolescent can reduce the risk of them misusing alcohol. Talk regularly with your adolescent one-on-one. Also try to eat dinner together as a family to facilitate communication. Ask your adolescent about topics that interest them and listen to them when they talk. Encourage them to express their opinions during everyday conversations.

Be aware that adolescents are often reluctant to talk about sensitive issues such as alcohol. Do not make all one-on-one time with your adolescent a time for serious discussion, as they may begin to avoid these situations. Avoid interrogating them, as this may cause them to be less open with you. Ask open-ended questions rather than ones that have simple “yes” or “no” answers. If you hear something you don’t like when talking to your adolescent, try not to respond with anger.

Be prepared to seek help to improve your parenting

Remember that it can be hard to be a parent. Do not hesitate to seek advice if you have difficulties with parenting. You should obtain advice from professional sources if you feel it would benefit your relationship or your communication with your adolescent.
